# Plasma Lipidomics Identifies Unique Lipid Signatures and Potential Biomarkers for Patients With Aortic Dissection

**DOI:** 10.3389/fcvm.2021.757022

**Published:** 2021-10-28

**Authors:** Huang Huang, Guozhu Ye, Song-qing Lai, Hua-xi Zou, Bin Yuan, Qi-cai Wu, Li Wan, Qun Wang, Xue-liang Zhou, Wen-jun Wang, Yuan-ping Cao, Jian-feng Huang, Shi-li Chen, Bi-cheng Yang, Ji-chun Liu

**Affiliations:** ^1^Department of Cardiac Surgery, The First Affiliated Hospital of Nanchang University, Nanchang, China; ^2^Center for Excellence in Regional Atmospheric Environment and Key Laboratory of Urban Environment and Health, Institute of Urban Environment, Chinese Academy of Sciences, Xiamen, China; ^3^Shanghai Key Laboratory of Biliary Tract Disease Research, Department of General Surgery, Xinhua Hospital Affiliated to Shanghai Jiao Tong University School of Medicine, Shanghai, China; ^4^Jiangxi Provincial Key Laboratory of Birth Defect for Prevention and Control, Jiangxi Provincial Maternal and Child Health Hospital, Nanchang, China

**Keywords:** aortic dissection, plasma, lipidomics, phospholipid, triglyceride, sphingolipid, fatty acid, biomarker

## Abstract

Aortic dissection (AD) is a catastrophic cardiovascular emergency with a poor prognosis, and little preceding symptoms. Abnormal lipid metabolism is closely related to the pathogenesis of AD. However, comprehensive lipid alterations related to AD pathogenesis remain unclear. Moreover, there is an urgent need for new or better biomarkers for improved risk assessment and surveillance of AD. Therefore, an untargeted lipidomic approach based on ultra-high-performance liquid chromatograph-mass spectrometry was employed to unveil plasma lipidomic alterations and potential biomarkers for AD patients in this study. We found that 278 of 439 identified lipid species were significantly altered in AD patients (*n* = 35) compared to normal controls (*n* = 32). Notably, most lipid species, including fatty acids, acylcarnitines, cholesteryl ester, ceramides, hexosylceramides, sphingomyelins, lysophosphatidylcholines, lysophosphatidylethanolamines, phosphatidylcholines, phosphatidylinositols, diacylglycerols, and triacylglycerols with total acyl chain carbon number ≥54 and/or total double bond number ≥4 were decreased, whereas phosphatidylethanolamines and triacylglycerols with total double bond number <4 accumulated in AD patients. Besides, the length and unsaturation of acyl chains in triacylglycerols and unsaturation of 1-acyl chain in phosphatidylethanolamines were decreased in AD patients. Moreover, lysophosphatidylcholines were the lipids with the largest alterations, at the center of correlation networks of lipid alterations, and had excellent performances in identifying AD patients. The area under the curve of 1.0 and accuracy rate of 100% could be easily obtained by lysophosphatidylcholine (20:0/0:0) or its combination with lysophosphatidylcholine (17:0/0:0) or lysophosphatidylcholine (20:1/0:0). This study provides novel and comprehensive plasma lipidomic signatures of AD patients, identifies lysophosphatidylcholines as excellent potential biomarkers, and would be beneficial to the pathogenetic study, risk assessment and timely diagnosis and treatment of AD.

## Introduction

Aortic dissection (AD), defined as the progressive separation of aortic wall layers, is a catastrophic cardiovascular emergency with an acute onset, a poor prognosis, and little preceding symptoms (1, 2). The incidence of thoracic AD is estimated to be 2.9–4.3 cases per 100,000 individuals per year, but deemed to be underestimated due to the large undiagnosed population (1). Notably, in patients with acute ascending AD who do not have surgery, ≥50% die within 48 h, and up to 90% die within 3 months (3). Moreover, even with surgical repair, the 24-h mortality rate for acute type A AD can reach 10%, 13% at 7 d, and about 20% at 30d (4). Therefore, research on AD pathogenesis and its diagnosis and treatment is of great importance and urgency.

Accumulating data indicate that abnormal lipid metabolism is closely related to the pathogenesis of AD (5–9). Lipid and atherosclerotic profiles revealed that total cholesterol, low density lipoprotein cholesterol, and apo A were significantly lower in patients with AD compared to patients with abdominal aortic aneurysms, even though less lipid lowering drugs were administrated (5). Targeted serum metabolomics discovered that the trimethylamine N-oxide level was significantly higher, whereas those of choline, betaine, and carnitine were significantly lower in patient with AD compared to normal controls, and that trimethylamine N-oxide had significant positive correlations with parameters on AD severity, including interleukin-6, D-dimer, C-reactive protein, and maximum aortic diameter on admission (6). Besides, the trimethylamine N-oxide level was significantly increased in AD patients with plaque rupture compared to AD patients without plaque rupture, and not affected by the incidence of hypertension (6). In addition, knockout of vascular smooth muscle cell-specific E-prostanoid receptor 4 gene induced AD in angiotensin II-infused mice with severe degradation of aortic elastic fiber, smooth muscle cell dedifferentiation, increased vascular NADPH oxidase 1 activity, reactive oxygen species generation, macrophage infiltration, matrix metalloproteinase-2/9 levels, and monocyte chemoattractant protein-1 expression, and higher blood pressure (7). *In vitro* investigations further showed that vascular smooth muscle cell-specific E-prostanoid receptor 4 gene deficiency significantly enhanced angiotensin II-triggered mesenteric arterial vasoconstriction, probably via the activation of intracellular calcium release in vascular smooth muscle cells (7). Moreover, phospholipases and unsaturated fatty acids (FAs) were also demonstrated to have vital roles in the pathogenesis of AD (8).

Owing to the diagnostic challenges, such as rapidly propagating pathology, non-specific signs, analogy with other acute conditions, non-discrete symptomatology, and lack of management infrastructure, untimely diagnosis and/or misdiagnosis of AD remain common, which could markedly deteriorate patient outcomes (10). Currently, only D-dimer was used as the clinically relevant biomarker in the condition of suspected AD, with a specificity of 47% and a sensitivity of 97% (11). As with many other diseases, the search for new or better markers is needed to improve the risk assessment and surveillance of AD, since the diagnostic specificity of available markers is insufficient (12). Lipids, accounting for nearly or more than 50% of the metabolome in many biological samples, are drawing more and more interests as potential biomarkers for the prediction of cardiovascular events (12). On the other hand, comprehensive lipid alterations related to the pathogenesis of AD remain unclear. Accordingly, an untargeted lipidomic approach based on ultra-high-performance liquid chromatograph-mass spectrometry was employed to discover the unique lipid signatures of patients with AD and relevant potential biomarkers in this study.

## Materials and Methods

### Subjects

After obtaining informed consent from all subjects and approval from the Ethics Committee of The First Affiliated Hospital of Nanchang University, the plasma samples were collected from 32 normal controls (healthy individuals) and 35 patients with AD. All patients with AD were suffering of an episode of chest and/or back pain lasting 5 min or longer within 3 d. Furthermore, we confirmed the diagnosis of AD according to computed tomography angiography, and further excluded the patients with Marfan syndrome, other connective tissue disorders or hemodialysis. The blood sample was collected immediately after the diagnosis of AD at the Department of Cardiovascular Surgery. Meanwhile, the normal controls were collected from the medical examination center, and excluded from those with atherosclerotic diseases, aneurysms, or valvular diseases. All the subjects fasted for at least 8 h prior to the blood sample collection. The blood sample was collected in the tube with EDTA, and immediately centrifuged at 1,500 g for 15 min at 4°C to prepare the plasma sample, which was stored at −80°C for subsequent preparation.

Demographic characteristics of the subjects were summarized in Supplementary Table 1. There were no significant differences in gender ratio (m/f, 18/14 vs. 23/12), age (52.0 ± 3.5 vs. 55.6 ± 11.1 y), and the incidence of chronic obstructive pulmonary disease (0 vs. 5.7%) between normal controls and patients with AD. Notably, the incidence of hypertension in patients with AD (82.9%) was significantly higher than that in normal controls (12.5%).

### Materials

HPLC-grade acetonitrile, methanol, and isopropanol were purchased from Merck (Germany). Distilled water was obtained from Watsons (Hong Kong). Ammonium acetate and methyl tert-butyl ether were gained from Sigma-Aldrich (USA).

### Sample Preparation

Following thawing on ice, the plasma sample was mixed thoroughly, and then 50 μL of the plasma sample was pipetted to a 2-mL centrifuge tube. Three hundred microliter of pre-cooled methanol, containing 0.32 μg/mL of sphingomyelin (SM, d18:1/12:0) and 0.25 μg/mL of FA 18:0-d3 as the internal standard in the positive and negative mode, respectively, was subsequently added to the sample, followed by 1-min vortex oscillation. Then, the sample was added with 1 mL of methyl tert-butyl ether, vortexed for 1 min, and gently vibrated for another 1 h. After that, the sample was added with 300 μL of water, and vortexed for 1 min. After equilibration at 4°C for 10 min, the sample was centrifuged at 14,000 g for 15 min. Two 400-μL aliquots of the upper lipid extract were separately pipetted into the new centrifuge tube, vacuum-dried, and then stored at −80°C. Finally, the dried lipid extract was dissolved with 120 μL of acetonitrile/isopropanol/water solution (v/v/v = 65:30:5) for the instrumental analysis in the negative ion mode. Meanwhile, 30 μL of the above dissolved sample was further diluted by 60 μL of the above-mentioned acetonitrile/isopropanol/water solution, and then used for the instrumental analysis in the positive ion mode.

To evaluate the analytical performance of the lipidomic approach, quality control samples were prepared by mixing equal parts of all plasma samples, and processed with the same parameters as the analytical sample during all processes involved in the sample preparation, instrumental analysis, and data processing.

### Instrumental Analysis

The plasma lipid profiling was acquired by a Q Exactive Plus high-resolution mass spectrometer (Thermo Scientific, USA) equipped with an Ultimate 3000 UHPLC system (Thermo Scientific, USA). Parameters on the chromatographic separation in the positive ion mode were the same as those in the negative ion mode. Five microliter of the dissolved sample was injected for the lipid separation by a BEH C_8_ column (100 × 2.1 mm, 1.7 μm, Waters Co., USA). The column temperature was 55°C. The lipids were eluted by the binary mobile phase A (acetonitrile/water solution, v/v = 6:4, containing 10 mM ammonium acetate) and B (isopropanol/acetonitrile solution, v/v = 9:1, containing 10 mM ammonium acetate). The flow rate was 0.26 mL/min. The elution gradient was conducted as follows: initial 32% B maintained for 1.5 min, linearly increased to 85% B from 1.5 to 15.5 min, to 97% B from 15.5 to 15.6 min, maintained at 97% B from 15.6 to 18.0 min, and then decreased to 32% B from 18.0 to 18.1 min, finally maintained at 32% B from 18.1 to 20.0 min. The temperature of the sample manager was 10°C.

The lipids eluted from the column were ionized by electrospray ionization in the mass spectrometer, and the mass signals were detected in full scan MS and -data dependent MS/MS (ddMS^2^) mode, with the resolution of 70,000 and 17,500, respectively. The spray voltage (kV) was +3.8 and −3.0 in the positive and negative ion mode, respectively. Other parameters were identical in the positive and negative ion mode, and set as follows: capillary temperature (°C), 320; aux gas heater temperature (°C), 350; sheath gas flow rate (arb), 35; aux gas flow rate (arb), 8; S-lens RF level, 50; mass scanning range (m/z), 100–1,500; TopN (N, the number of the fragmentation ions with the highest abundance), 10; stepped normalized collision energy (NCE), 25, 35, and 45%.

### Data Pre-processing

A peak table containing the retention time, m/z, peak area, and lipid identification results was obtained by peak matching and structural identification using the MS-DIAL software (13). Briefly, the raw MS data were converted to the common file format of Reifycs Inc. (.abf) using the Reifycs ABF converter. After that, MS-DIAL software was used for feature detection, spectra deconvolution, lipid identification, and peak alignment among samples. MS/MS spectra-based lipid identification was performed in MS-DIAL software by searching the acquired MS/MS spectra against the internal *in silico* MS/MS spectra database. It includes MS1 and MS/MS information of common lipid species. Tolerances for MS1 and MS/MS searches were set to 0.01 and 0.05 Da, respectively. Data collected in the positive ion mode were normalized to the internal standard SM (d18:1/12:0), and then multiplied by 8 × 10^8^. Meanwhile, data collected in the negative ion mode were normalized to the internal standard FA 18:0-d_3_, and then then multiplied by 1 × 10^10^. After that, data from the positive and negative ion mode were combined and defined as relative abundances of lipids, which were then used for subsequent statistical analysis.

### Statistical Analysis

Chi-square test was done using PASW Statistics 18 (SPSS Inc., USA). Following unit variance scaling, principal component analysis, partial least square discriminant analysis, classical univariate receiver operating characteristic curve analysis, multivariate exploratory receiver operating characteristic curve analysis, and support vector machine algorithm for the feature ranking and sample classification were performed by MetaboAnalyst 5.0 (14). Two-tailed Mann-Whitney U test was carried out via MultiExperiment Viewer 4.9.0 (15). After unit variance scaling, the data were used for heat map plot using MultiExperiment Viewer 4.9.0. Correlation networks of the lipid signatures were constructed using Cytoscape 2.8.2 (16). Spearman correlation analysis was performed by MATLAB (MathWorks Inc., USA). The level of significance was 0.05, and further corrected by the false discovery rate employing Benjamini-Hochberg correction via MultiExperiment Viewer 4.9.0.

## Results

### Substantial Changes in the Lipid Profile of Patients With AD

**Figure 1:**
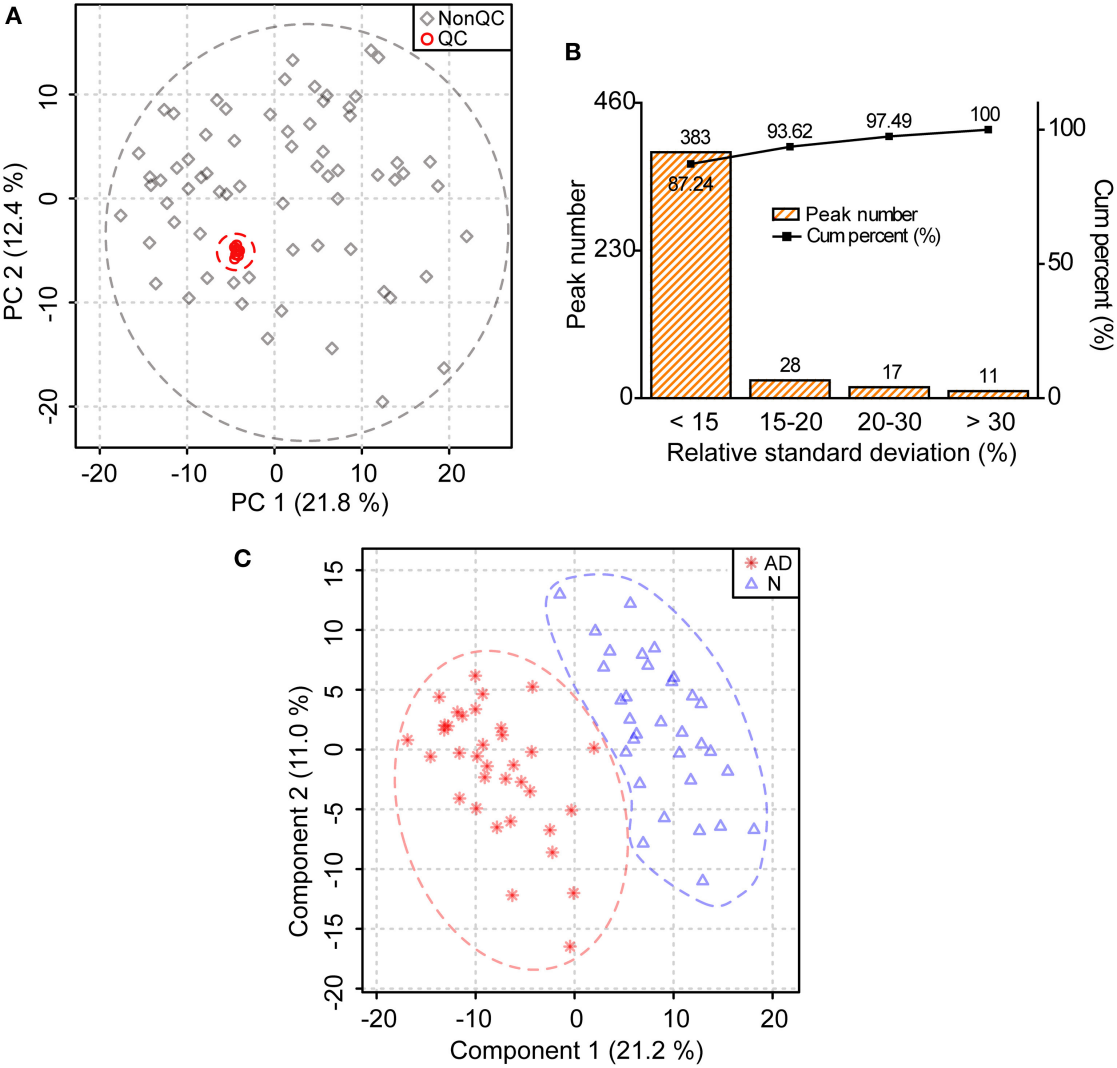
Substantial changes in the lipid profile of patients with AD. **(A)** Distribution of quality control (QC) samples in the score plot of principal component analysis. The explained variances are provided in brackets. **(B)** Distribution of relative standard deviations of ions in QC samples. **(C)** Changes in the lipid profile of patients with AD in the score plot of partial least square discriminant analysis. The explained variances are provided in brackets. *n* = 32 and 35 in the normal (N) and AD group, respectively.

The score plot of principal component analysis showed that 10 quality control samples were located closely to each other (Figure 1A). Besides, it was clear from the relative standard deviation distribution that among the 439 lipids identified, there were 383, 411, and 428 lipids, separately accounting for 87.24, 93.62, and 97.49%, with relative standard deviations <15, 20, and 30%, respectively (Figure 1B). Above data demonstrated that the lipidomic approach was highly repeatable, stable, and reliable in this study (17, 18). Furthermore, we found that the lipid profile of patients with AD differed greatly from that of normal controls in the score plot of partial least square discriminant analysis, indicating substantial changes in lipid metabolism in patients with AD (Figure 1C).

### Characteristic Lipid Metabolism in Patients With AD

**Figure 2:**
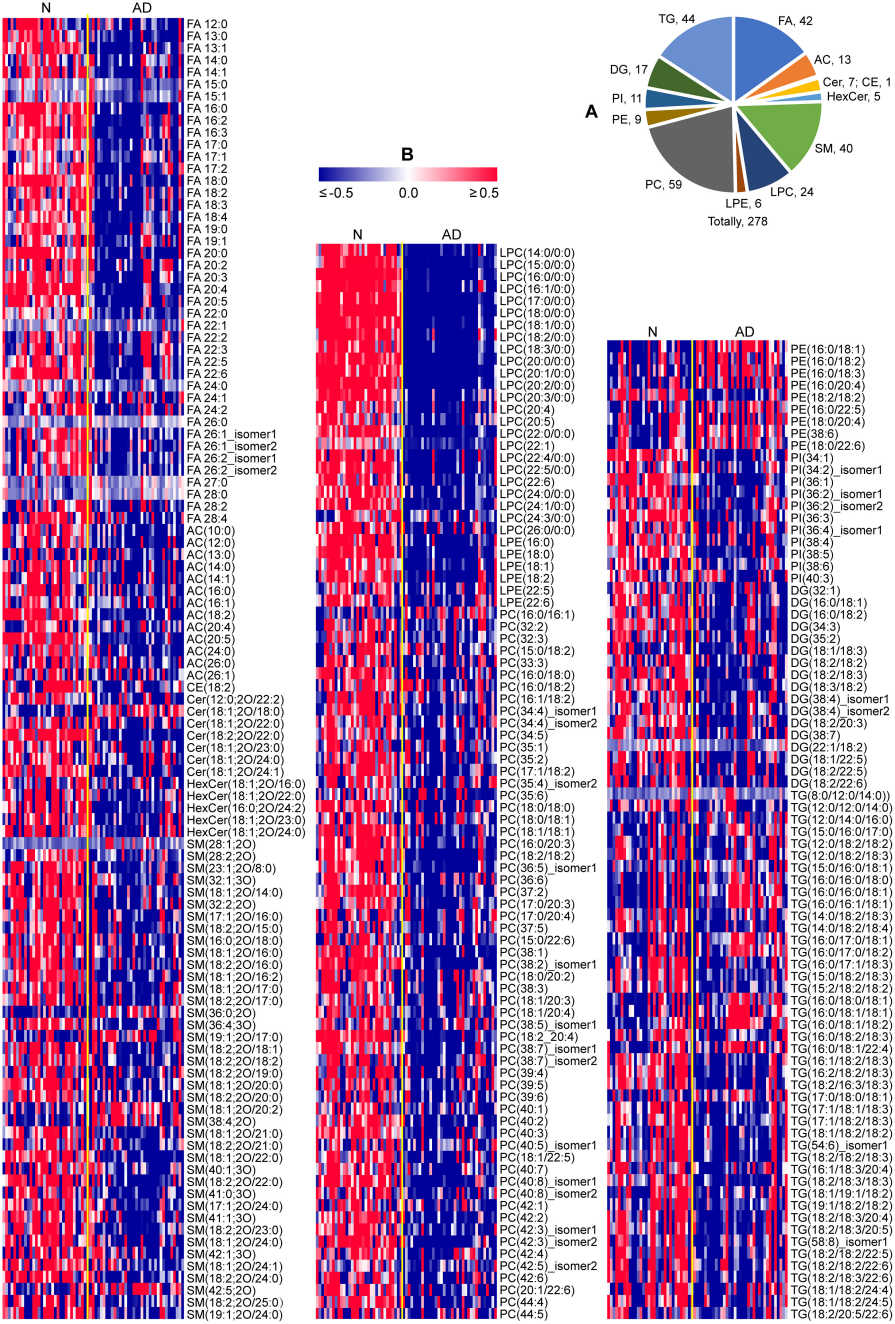
Characteristic lipid metabolism in patients with AD. **(A)** Pie chart indicating the number of lipids significantly altered in patients with AD (*P* < 0.05, two-tailed Mann-Whitney *U* test). **(B)** Heat map plot of lipid alterations in patients with AD. Red/blue color: high/low abundance. *n* = 32 and 35 in the normal (N) and AD group, respectively. FA, fatty acid; AC, acyl carnitine; CE, cholesteryl ester; Cer, ceramide; HexCer, hexosylceramide; SM, sphingomyelin; LPC, lysophosphatidylcholine; LPE, lysophosphatidylethanolamine; PC, phosphatidylcholine; PE, phosphatidylethanolamine; PI, phosphatidylinositol; DG, diglyceride; TG, triglyceride.

**Figure 3:**
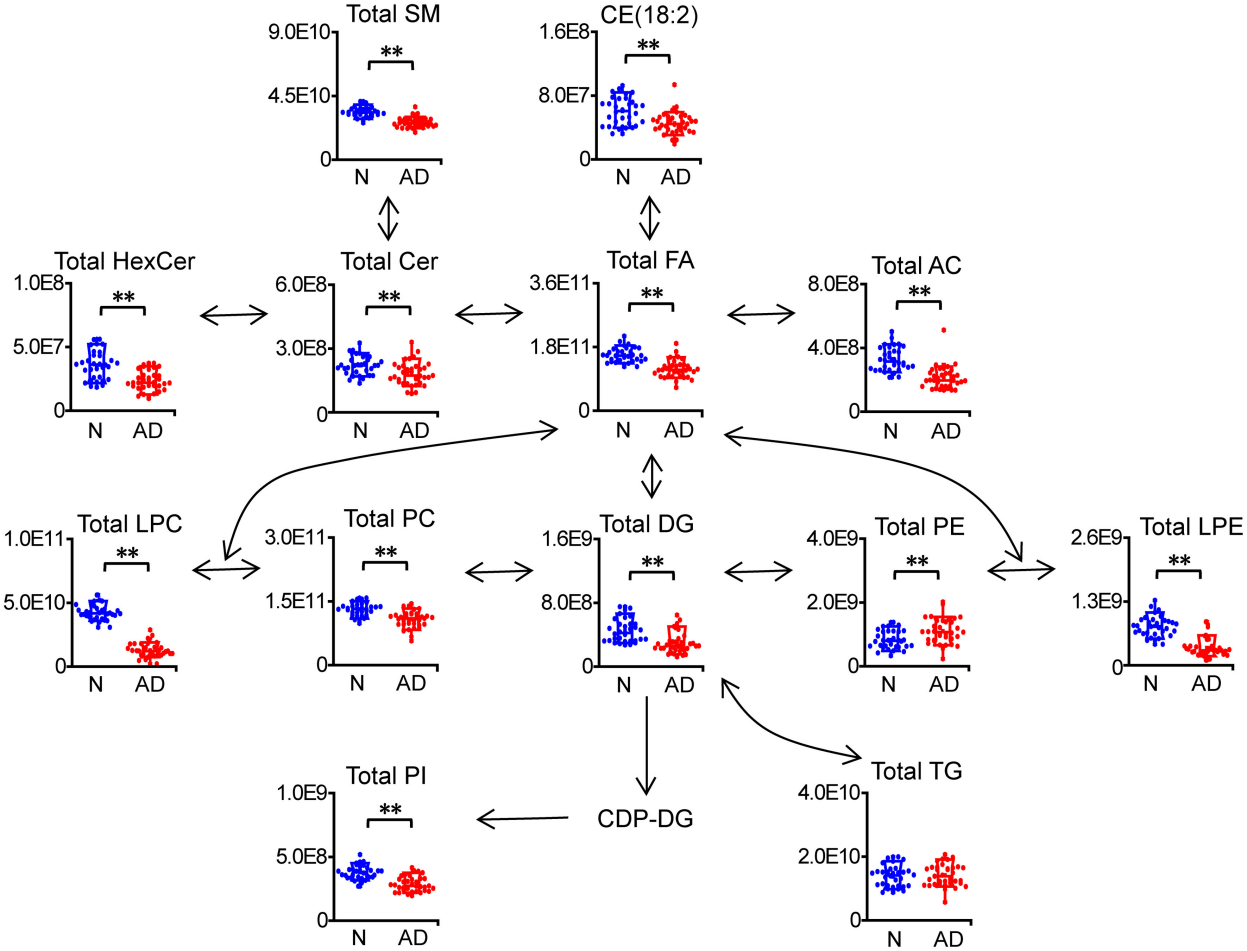
Characteristic changes in total lipids of each category in patients with AD. *n* = 32 and 35 in the normal (N) and AD group, respectively. **P < 0.01, two-tailed Mann-Whitney *U* test. The relative abundances of lipids were used for the box plot. Arrows indicate the conversion between lipids. FA, fatty acid; AC, acyl carnitine; CE, cholesteryl ester; Cer, ceramide; HexCer, hexosylceramide; SM, sphingomyelin; LPC, lysophosphatidylcholine; LPE, lysophosphatidylethanolamine; PC, phosphatidylcholine; PE, phosphatidylethanolamine; PI, phosphatidylinositol; DG, diglyceride; TG, triglyceride.

In total, 278 of 439 identified lipid species were found to be significantly altered in patients with AD compared to normal controls, including 42 FAs, 13 acylcarnitines (ACs), 1 cholesteryl ester (CE), 7 ceramides (Cers), 5 hexosylceramides (HexCers), 40 SMs, 24 lysophosphatidylcholines (LPCs), 6 lysophosphatidylethanolamines (LPEs), 59 phosphatidylcholines (PCs), 9 phosphatidylethanolamines (PEs), 11 phosphatidylinositols (PIs), 17 diacylglycerols (DGs), and 44 triacylglycerols (TGs) (Figure 2A; Supplementary Table 2). Heatmap plot showed that levels of most lipids were significantly decreased, including FAs, ACs, CE, Cers, HexCers, SMs, LPCs, LPEs, PCs, PIs, DGs, and TGs with the total number of carbons in the acyl chains ≥54, while levels of most PEs were significantly increased in patients with AD compared to those in normal controls (Figure 2B; Supplementary Table 2). Consistently, total levels of FAs, ACs, CE, Cers, HexCers, SMs, LPCs, LPEs, PCs, PIs, and DGs were significantly decreased, while the total level of PEs were significantly increased in patients with AD compared to those in normal controls (Figure 3; Supplementary Table 3). However, significant changes in the total level of TGs were not observed in patients with AD compared to normal controls, which could be due to the phenomena that changes in TGs with the total carbon number of acyl chains <54 were different from those in TGs with the total carbon number of acyl chains ≥54 in patients with AD (Figure 3; Supplementary Table 3). Above data demonstrated decreases in most lipid species and accumulation of PEs in the plasma of patients with AD compared to normal controls, which suggested that most lipid species might accumulate, while PEs might decrease in the dissection of patients with AD compared to normal controls.

### Characteristic Changes in the Acyl Chains of TGs in Patients With AD

**Figure 4:**
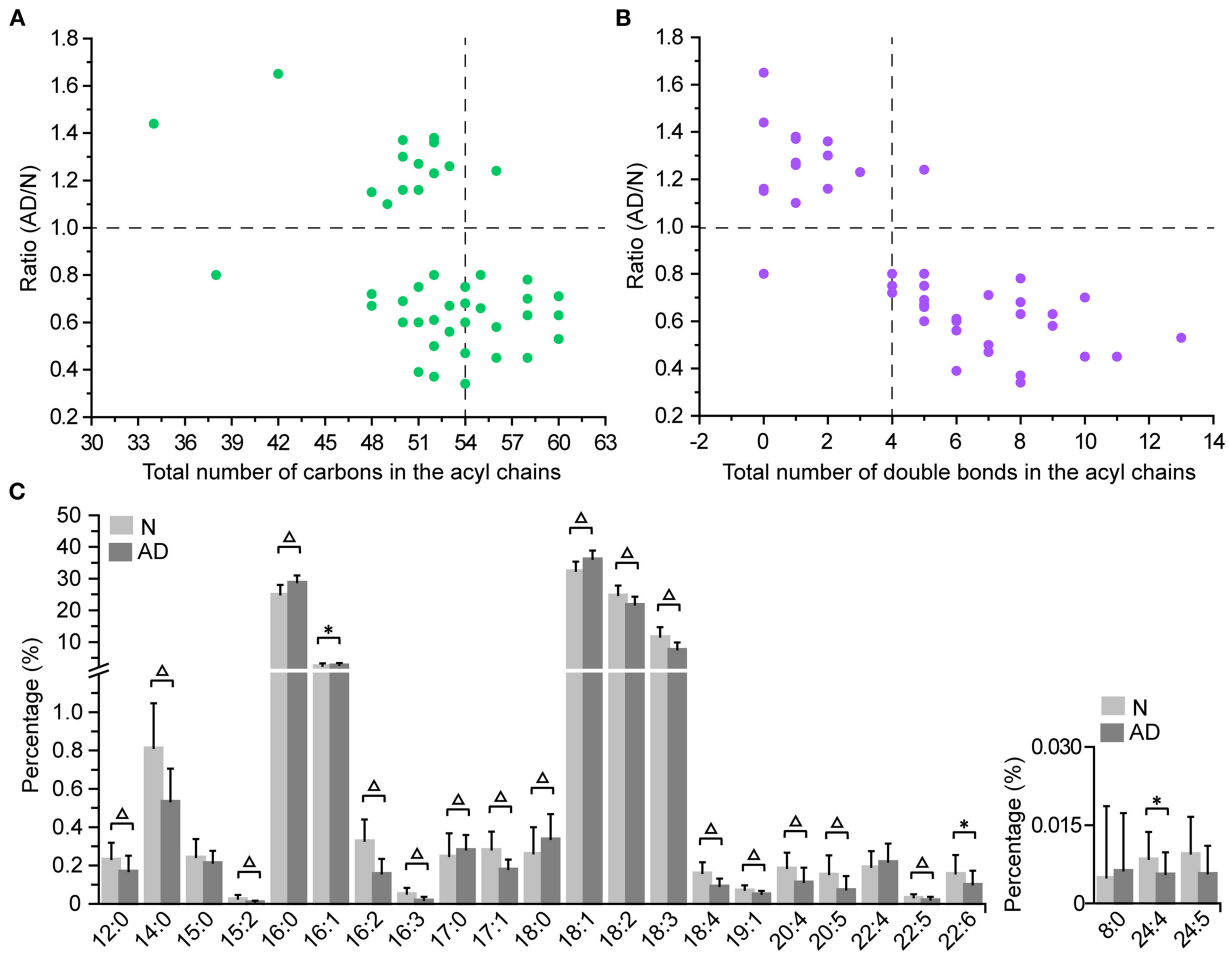
Characteristic changes in the acyl chains of TGs in patients with AD. Changes in the total number of carbons **(A)** and double bonds **(B)** in the acyl chains of TGs. **(C)** Changes in the percentage of the acyl chains of TGs. The column denotes the mean plus standard deviation. n = 32 and 35 in the normal (N) and AD group, respectively. *P < 0.05; Δ*P* < 0.01, two-tailed Mann-Whitney *U* test.

Since changes in TGs with the total carbon number of acyl chains <54 were different from those in TGs with the total carbon number of acyl chains ≥54, we further investigated changes in the composition and percentage of the acyl chains in TGs. It was clear from the total carbon distribution in the acyl chains of TGs that most TGs with the total carbon number of acyl chains ≥54 were significantly decreased in patients with AD, which was consistent with the results observed in Figures 2, 4A; Supplementary Table 2. In addition, it could be clearly observed from the double bond distribution in the acyl chains of TGs that most TGs with the total number of double bonds in the acyl chains ≥4 were significantly decreased, while most TGs with the total number of double bonds in the acyl chains <4 were significantly increased in patients with AD (Figure 4B). Moreover, from the changes in the percentage of the acyl chains in TGs, we could observe that polyunsaturated FA chains (the number of double bonds ≥2, i.e., 15:2, 18:2, 20:4, 22:5, 24:4) and FA chains with carbon number >18 (i.e., 19:1, 20:4, 20:5, 22:5, 22:6) were significantly decreased, while 16:0, 16:1, 17:0, 18:0, and 18:1 were significantly increased in patients with AD (Figure 4C; Supplementary Table 4). These data demonstrated decreases in the length, polyunsaturation, and total number of carbons in the acyl chains of TGs in the plasma of patients with AD.

### Characteristic PE Accumulation and Its Acyl Chain Alterations in Patients With AD

**Figure 5:**
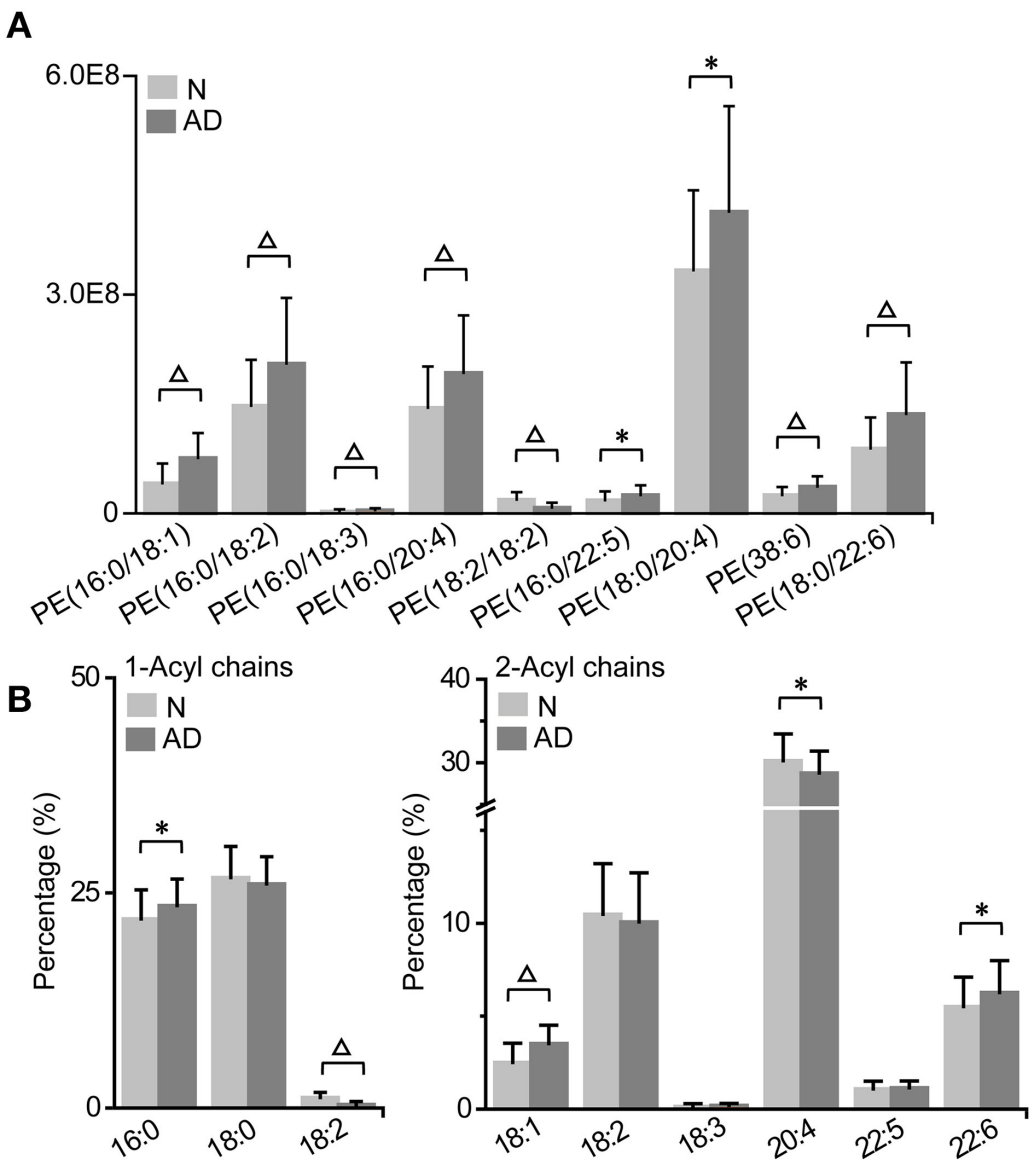
Characteristic PE accumulation **(A)** and its acyl chain alterations **(B)** in patients with AD. The column denotes the mean plus standard deviation. *n* = 32 and 35 in the normal (N) and AD group, respectively. *P < 0.05; Δ*P* < 0.01, two-tailed Mann-Whitney *U* test. **(A)** The relative abundances of lipids were used for the histogram.

We found that most PE accumulated, including PE (16:0/18:1), PE (16:0/18:2), PE (16:0/18:3), PE (16:0/20:4), PE (16:0/22:5), PE (18:0/20:4), PE (38:6), and PE (18:0/22:6), while PE (18:2/18:2) was decreased in patients with AD (Figure 5A; Supplementary Table 2). Consistently, the total PE level was also increased in patients with AD (Figure 3; Supplementary Table 3). Subsequently, characteristic changes in the acyl chains of PEs in patients with AD were further examined (Figure 5B; Supplementary Table 5). It was showed that saturated acyl chains were only located at the sn-1 position of the glycerol moiety, and that unsaturated acyl chains were mainly distributed at the sn-2 position of the glycerol moiety. Additionally, the percentage of 16:0 at the sn-1 position was significantly increased, while that of 18:2 at the sn-1 position was significantly decreased, which indicated an increase in the saturation at the sn-1 position of PEs in patient with AD. Moreover, the percentages of 18:1 and 22:6 at the sn-2 position were significantly increased, while that of 20:4 at the sn-2 position was significantly decreased, indicating significant acyl chain remodeling at 2-acyl chains of PEs in patients with AD.

### Correlation Networks of Lipid Alterations in Patients With AD

**Figure 6:**
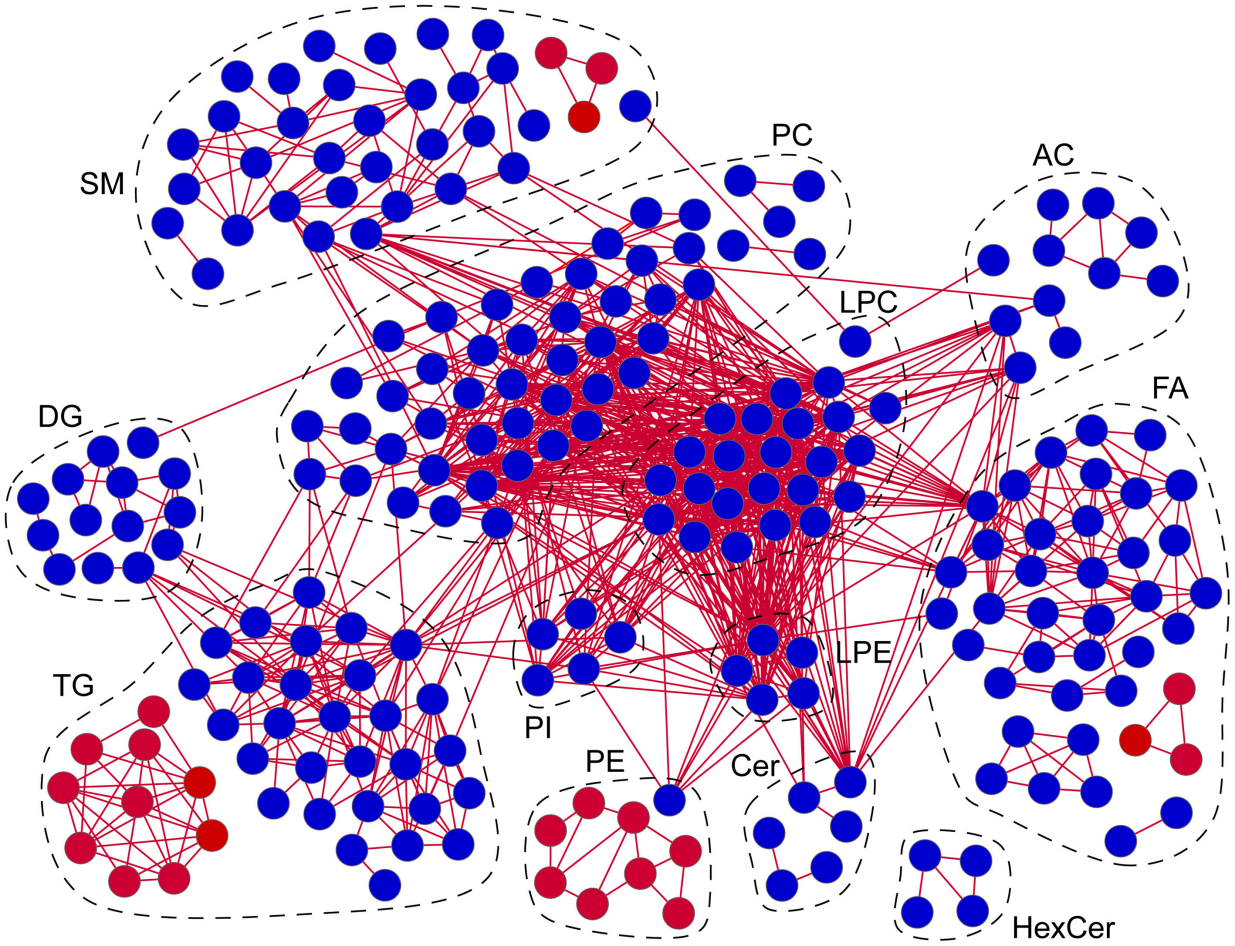
Correlation networks of lipid alterations in patients with AD. Red/blue circles: lipids significantly increased/decreased in patients with AD. Red lines: positive correlations. Absolute values of Spearman correlation coefficients were set to be higher than 0.75. *n* = 67. FA, fatty acid; AC, acyl carnitine; Cer, ceramide; HexCer, hexosylceramide; SM, sphingomyelin; LPC, lysophosphatidylcholine; LPE, lysophosphatidylethanolamine; PC, phosphatidylcholine; PE, phosphatidylethanolamine; PI, phosphatidylinositol; DG, diglyceride; TG, triglyceride.

To determine the latent relationships between lipid alterations in patients with AD, correlation networks were further constructed with the standard that the absolute values of Spearman correlation coefficients were >0.75 (Figure 6). Totally, 1,086 edges and 240 lipids (including 40 FAs, 11ACs, 6 Cers, 4 HexCers, 35 SMs, 24 LPCs, 5 LPEs, 48 PCs, 9 PEs, 5 PIs, 14 DGs, and 39 TGs) were retained in the correlation networks. Besides, the correlation coefficients between lipids in the correlation networks were all positive. Moreover, it was clear that LPCs and PCs were located at the center of the correlation networks, suggesting potential pivotal roles in the molecular pathogenesis of AD. These data demonstrated the high correlations among lipid alterations and potential involvement of lipids in the molecular pathogenesis of AD, especially LPCs and PCs.

### Potential Biomarkers for the Identification of Patients With AD

**Figure 7:**
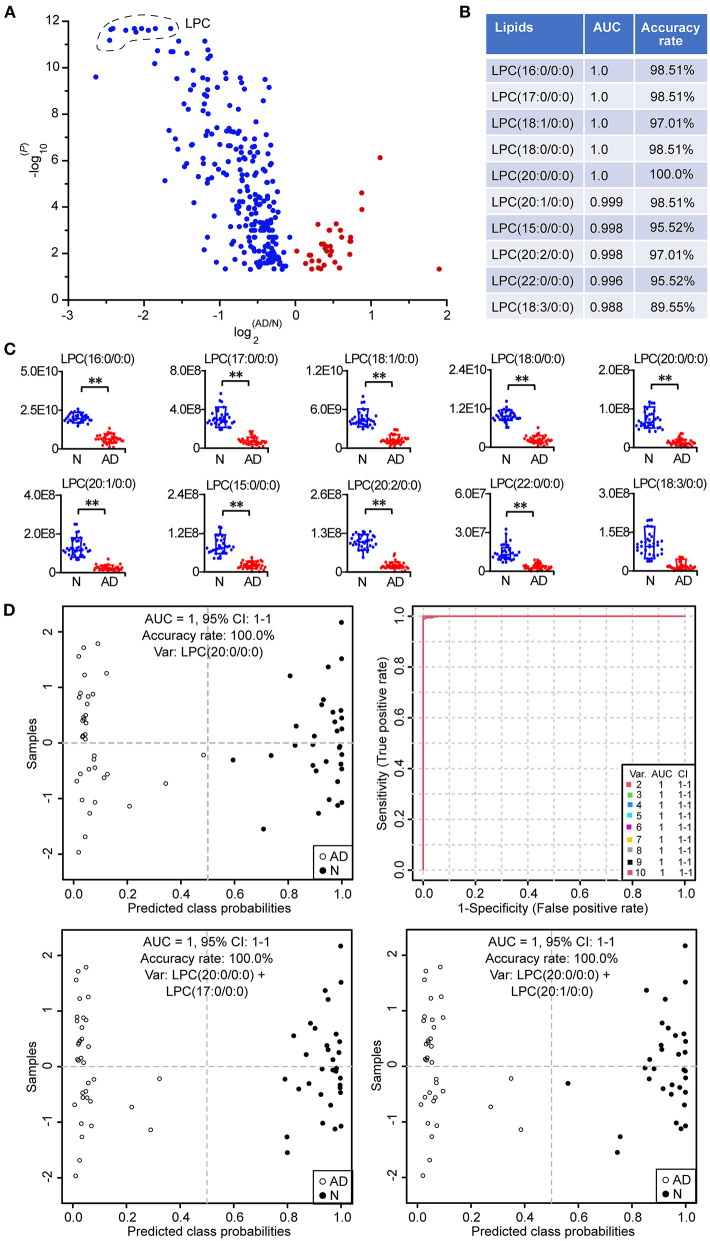
Potential biomarkers for the identification of patients with AD. **(A)** Volcano plot of lipid alterations. Only the differential lipids (*P* < 0.05, two-tailed Mann-Whitney *U* test) are shown in the plot. **(B)** Classical univariate ROC (receiver operating characteristic) curve analyses of the top 10 lipids with the lowest *P*-value. **(C)** Box plot of the top 10 lipids with the lowest *P*-value. The relative abundances of lipids were used. **P < 0.01, two-tailed Mann-Whitney U test. **(D)** Discovery of important potential biomarkers by multivariate ROC curve analyses and the performance. The predicted class probability of each sample was obtained from the 100 cross-validations using the support vector machine algorithm. The cutoff value of probabilities for the sample classification was 0.5. Relevant potential biomarkers used for the sample classification were provided in the graph. LPC, lysophosphatidylcholine. *n* = 32 and 35 in the normal (N) and AD group, respectively. AUC, area under the curve; CI, confidence interval.

The volcano plot showed that LPCs were the lipids with the largest alterations according to the *P*-value, since the top 10 lipids with the lowest *P*-values were all LPCs, including LPC (20:0/0:0), LPC (17:0/0:0), LPC (18:0/0:0), LPC (18:1/0:0), LPC (16:0/0:0), LPC (20:1/0:0), LPC (20:2/0:0), LPC (15:0/0:0), LPC (22:0/0:0), and LPC (18:3/0:0) (Figure 7A; Supplementary Table 2). Subsequently, the above 10 LPCs were used separately as the potential biomarker for the classical univariate receiver operating characteristic curve analyses and identification of patients with AD based on the support vector machine algorithm (Figure 7B). The results showed that when LPC (20:0/0:0), LPC (17:0/0:0), LPC (18:0/0:0), LPC (18:1/0:0), LPC (16:0/0:0), LPC (20:1/0:0), LPC (20:2/0:0), LPC (15:0/0:0), and LPC (22:0/0:0) were used as the potential biomarker, respectively, the area under the curve could be above 0.99, and the accuracy rate could be above 95%, demonstrating that above 9 LPCs had excellent diagnostic performances in identifying patients with AD from normal controls. It was clear from the box plot that the above 10 LPCs were all significantly decreased in patients with AD (Figure 7C). Although 100% of both patients with AD and normal controls could be correctly identified by LPC (20:0/0:0) alone, the predicted class probability of one patient with AD (0.48) was close to the classification threshold (0.5) (Figure 7D). To improve the classification performance, the multivariate exploratory receiver operating characteristic curve analysis based on the support vector machine algorithm was used for further feature selection from the above 10 LPCs and relevant sample classification. It was revealed that when selecting 2–10 feature variables to identify patients with AD, LPC (20:0/0:0), LPC (17:0/0:0), and LPC (20:1/0:0) were screened as the feature variables with the highest frequency among the 10 LPCs, and the value of each area under the curve could reach 1.0. Therefore, LPC (17:0/0:0) and LPC (20:1/0:0) were separately combined with LPC (20:0/0:0) as the biomarker combination to distinguish patients with AD from normal controls. It was showed that patients with AD could be more clearly distinguished from normal controls by the above combinatorial biomarkers compared to the single biomarker LPC (20:0/0:0), especially the combination of LPC (20:0/0:0) and LPC (17:0/0:0). These data demonstrated that LPCs owned excellent diagnostic performances in discriminating patients with AD from normal controls.

## Discussion

FAs are located at the metabolic center of lipids, which can interconvert with other lipids with acyl chains, provide energy through oxidative degradation, and act as signaling molecules to mediate many pathophysiological processes. Significant decreases in most plasma FAs (including saturated, monounsaturated, and polyunsaturated FAs) and ACs in patients with AD in this study suggested potential disorders of lipolysis, FA synthesis, transport and/or oxidation in patients suffering from AD. It was found that the plasma level of superoxide dismutase was decreased, while that of malondialdehyde was increased in patients with AD compared to normal controls (19). Consistently, proteomics and western blotting investigations showed that the malondialdehyde level was increased, while levels of total, Cu/Zn^−^, and extracellular superoxide dismutase were decreased in the aortic tissue of patients with AD compared to normal controls (20). It was revealed that melatonin treatment increased levels of superoxide dismutase, sirtuin 1, and nuclear factor erythroid 2-related factor 2, decreased the malondialdehyde level, and prevented the deterioration of AD in β-aminopropionitrile fumarate-treated mice, evinced by decreases in the incidence, aneurysmal dilation and vascular stiffness, improvement of aortic morphology, and the inhibition of matrix metalloproteinase expression, elastin degradation, macrophage infiltration, oxidative stress damages and vascular smooth muscle cell loss, and that suppressing sirtuin 1 signaling decreased the protective effects of melatonin on AD (21). Above data indicated increases in oxidative stress and polyunsaturated FA peroxidation, and a decrease in the antioxidant capacity in patients with AD compared to normal controls, and that decreases in polyunsaturated FAs might be related to the increased oxidative degradation (22). Notably, the metabolism of polyunsaturated FAs in oxidative states would produce some lipid signaling mediators, such as prostaglandins, thus affecting the pathogenesis of AD via mediating molecular processes related to inflammation and oxidative stress (7, 23). Moreover, 13 odd-chain FAs, averagely accounting for 1.18% of total FAs in all the subjects, were found to be significantly decreased in AD patients in this study. Changes in odd-chain FAs and some lipids containing odd-chain FAs, such as LPC (17:0), CE (17:0), and monoacylglycerol (15:0), were discovered to be significantly associated with the incidence of ischemic heart disease and type 2 diabetes (24, 25).

We observed decreases in the percentages of 18:2 sn-1 and 20:4 sn-2 in PEs and polyunsaturated FA chains in TGs (such as 18:2, 20:4, 22:5, and 24:4), and increases in the percentages of 18:1 and 22:6 in PEs patients with AD in this study, which indicated the involvement of the unsaturated acyl chains of PEs and TGs in the pathogenesis of AD. Phospholipases and lysophospholipid acyltransferases mediate the deacylation and acylation, respectively, thus modulating the composition and percentage of acyl chains in phospholipids and related alterations in biological functions (26, 27). It was revealed that secreted phospholipase A2 group V from endothelial cells in the angiotensin II-treated aorta of mice could mobilize linoleate and oleate, which could ameliorate endoplasmic reticulum stress, and enhance the expression of lysyl oxidase, thus stabilizing the extracellular matrix, and that dietary linoleate or oleate supplementation abolished the susceptibility to AD in secreted phospholipase A2 group V-deficient mice (8). On the other hand, lysophosphatidylcholine acyltransferase 3 could enhance contents of phospholipids containing polyunsaturated FAs, and mediate arachidonic acid remodeling among different lipid species and eicosanoid release in macrophages, thus affecting the pathogenesis of cardiovascular diseases (27–29). Moreover, serum lipidome found that 3 DGs (including 18:0/18:2, 18:1/18:2, and 18:2/18:2) and 7 TGs (including 16:0/18:1/18:2, 16:0/18:2/18:2, 18:0/18:2/18:2, 18:1/18:1/18:2, 18:1/18:2/18:2, 18:2/18:2/18:2, and 18:2/18:2/20:4), all containing 18:2, were significantly correlated with the incidence of abdominal aortic aneurysm in human, and that the combination of above DGs and TGs with traditional risk factors significantly improved the diagnosis of abdominal aortic aneurysm compared to traditional risk factors alone (30). Besides, the increases in the percentages of short and saturated fatty acyls in TGs were correlated with elevated cardiovascular diseases (31).

Notably, we found that LPCs were the lipids with the largest alterations in the plasma lipidome of patients with AD, and located at the center of metabolic correlation networks, and that LPCs were demonstrated to be excellent potential biomarkers for identifying patients with AD in this study. LPCs can be acylated and deacylated by lysophospholipid acyltransferases and phospholipases to generate PCs and release FAs, respectively, thus affecting the pathogenesis of AD. Additionally, LPCs can acts as the ligands to activate peroxisome proliferator-activated receptors, which are commonly used as the targets for treating metabolic diseases, such as diabetes and cardiovascular diseases (32, 33). The decreases in LPCs in patients with AD in this study were consistent with their mediatory roles in the expression of peroxisome proliferator-activated receptors (31). Besides, it was discovered that levels of LPCs (including 16:0, 18:0, and 18:1) and lipoprotein-associated phospholipase A_2_ expression were increased in human atherosclerotic plaques, and that high levels of LPCs (16:0, 18:0, and 18:1) were correlated with increases in tumor necrosis factor-α, interleukin-1β, interleukin 6, macrophage inflammatory protein-1β, monocyte chemoattractant protein-1, macrophages, and lipids, and decreases in smooth muscle cells in human atherosclerotic plaques (34). Moreover, serum metabolomics discovered that a total of 8 LPCs, including LPC (16:0), LPC (16:1), LPC (18:0), LPC (18:1), LPC (18:2), LPC (18:3), LPC (20:3), and LPC (22:6), were significantly decreased in patients with AD (Stanford type A or B) compared to normal controls, and that levels of above LPCs did not vary with gender or blood pressure in patients with AD (Stanford type A or B) (9). However, the diagnostic performance of LPCs as potential markers for the identification of AD patients, such as the sensitivity, specificity, area under the curve, and accuracy rates, were not conducted in the study (9). Large-scale blood metabolomic profiling identified negatively strong associations of LPC (18:1) and LPC (18:2) with the risk of cardiovascular diseases in an age-dependent manner, stronger negative associations in older individuals (35). Decreases in LPCs in this study indicated potential disorders of phospholipid/lysophospholipid lipolysis, lysophospholipid acylation, and/or relevant lipid transport in AD patients. However, the specific role of LPCs in the pathogenesis of AD still needs further functional experiments to discover and verify.

Significant decreases in most plasma sphingolipids, including SMs, Cers, and HexCers, in patients with AD were also observed in this study, which indicated potential disorders of sphingolipid synthesis, degradation, and/or transport in the pathogenesis of AD (36). SMs, as the most abundant sphingolipid in plasma and lipoproteins, accumulated in both lipoproteins and atherosclerotic plaques (36, 37). Sphingomyelinase in atherosclerotic plaques could induce aggregation of low density lipoproteins enriched in Cers via hydrolysis of SMs, thus resulting in the initiation and progression of atherosclerosis (37). Moreover, both SMs and Cers are emerging as promising predictive and/or prognostic biomarkers for the diagnosis of many metabolic diseases, such as cardiovascular diseases (36).

In summary, we provided a comprehensive and novel presentation on plasma lipidomic signatures of patients with AD, which were characterized by decreases in most FAs, ACs, CE, Cers, HexCers, SMs, LPCs, LPEs, PCs, PIs, DGs, and TGs (with the total carbon number of acyl chains ≥54 and/or the total number of double bonds in the acyl chains ≥4), accumulation of PEs and triacylglycerols with the total number of double bonds in the acyl chains <4, and decreases in the length and unsaturation of acyl chains in TGs and unsaturation of 1-acyl chain in PEs. Additionally, pivotal pathophysiological processes and potential therapeutic targets related to above lipidomic signatures of AD were described and discussed. Moreover, LPCs were demonstrated to be useful as potential biomarkers for identifying AD, an accuracy of >95% could easily achieved with a single LPC. The results indicate that verifying the applicability of LPCs as suitable markers for the identification of AD using large-scale samples from multi-centers has a good prospect, which would be of great benefit to improving the prediction, diagnosis, and/or prognosis of AD.

## Data Availability Statement

The original contributions presented in the study are included in the article/Supplementary Material, further inquiries can be directed to the corresponding author/s.

## Ethics Statement

The studies involving human participants were reviewed and approved by the Ethics Committee of the First Affiliated Hospital of Nanchang University. The patients/participants provided their written informed consent to participate in this study. Written informed consent was obtained from the individual(s) for the publication of any potentially identifiable images or data included in this article.

## Author Contributions

HH, GY, S-qL, S-lC, B-cY, and J-cL conceived and designed this study. H-xZ, BY, Q-cW, LW, QW, X-lZ, W-jW, Y-pC, and J-fH collected the written informed consent and plasma samples. GY carried out the lipidomic data analysis, interpreted the data, wrote, and revised the manuscript. S-lC conducted the sample preparation, instrumental analysis, and data pre-processing for the lipidomic approach. HH, S-qL, B-cY, and J-cL checked the manuscript. All authors contributed to the article and approved the submitted version.

## Funding

This work was supported by the National Natural Science Foundation of China (Nos. 81960059 and 21705108), the Natural Science Foundation of Jiangxi, China (No. 20192BAB215003), the Academic and Technical Leader Plan of Jiangxi Provincial Main Disciplines (No. 20204BCJL23056), the Shanghai Pujiang Program (No. 17PJ1406100), and the Superior scientific and technological innovation team of Jiangxi Province (No. 20181BCB24014).

## Conflict of Interest

The authors declare that the research was conducted in the absence of any commercial or financial relationships that could be construed as a potential conflict of interest.

## Publisher's Note

All claims expressed in this article are solely those of the authors and do not necessarily represent those of their affiliated organizations, or those of the publisher, the editors and the reviewers. Any product that may be evaluated in this article, or claim that may be made by its manufacturer, is not guaranteed or endorsed by the publisher.
