# Supplementary material for: Plasma Lipidomics Identifies Unique Lipid Signatures and Potential Biomarkers for Patients With Aortic Dissection
Source: Front Cardiovasc Med. 2021 Oct 28;8:757022. doi: 10.3389/fcvm.2021.757022 (PMC8581228; doi:10.3389/fcvm.2021.757022)
Supplement: Supplementary file 1 [file Table_1.DOCX]

***Supplementary Material***

**Supplementary Table 1.** Baseline characteristics of the subjects.

| **Demographic Information** | **Normal Controls**  **(n = 32)** | **AD**  **(n = 35)** | ***P* value** |
| --- | --- | --- | --- |
| Gender (m/f) | 18/14 | 23/12 | 0.427 |
| Age (Years) | 52.0±3.5 | 55.6±11.1 | 0.204 |
| **Hypertension** | **4 (12.5%)** | **29 (82.9%)** | **8.72E9** |
| Chronic obstructive pulmonary disease | 0 (0%) | 2 (5.7%) | 0.17 |

Red bold fonts: significantly increased in patients with AD. The differences in gender ratio, and the incidence of hypertension and chronic obstructive pulmonary disease between normal controls and patients with AD were evaluated by the Chi-square test. Besides, the difference in age between normal controls and patients with AD were evaluated by the two-tailed Mann-Whitney U test.

**Supplementary** **Table 2.** Characteristic lipid metabolism in patients with AD.

| **Lipids** | **Adduct type** | **m/z**  **(Average)** | **m/z**  **(Expected)** | **AD/N** | ***P*** | **Adj. *P*** |
| --- | --- | --- | --- | --- | --- | --- |
| Fatty acid metabolism | | | | | | |
| FA 12:0 | [M-H2O-H]- | 199.16942 | 199.17039 | **0.81** | **0.001** | **0.003** |
| FA 13:0 | [M-H]- | 213.18488 | 213.186 | **0.70** | **2.16E-8** | **2.02E-7** |
| FA 13:1 | [M-H]- | 211.16933 | 211.17039 | **0.63** | **8.35E-10** | **1.08E-8** |
| FA 14:0 | [M-H]- | 227.20088 | 227.20171 | **0.84** | **0.004** | **0.008** |
| FA 14:1 | [M-H]- | 225.1857 | 225.186 | **0.62** | **7.42E-6** | **3.05E-5** |
| FA 15:0 | [M-H]- | 241.21634 | 241.2173 | **0.84** | **0.002** | **0.005** |
| FA 15:1 | [M-H]- | 239.20076 | 239.20171 | **0.85** | **0.014** | **0.027** |
| FA 16:0 | [M-H]- | 255.23222 | 255.233 | **0.76** | **3.20E-10** | **4.84E-9** |
| FA 16:2 | [M-H]- | 251.20184 | 251.20171 | **0.61** | **4.51E-7** | **2.61E-6** |
| FA 16:3 | [M-H]- | 249.18597 | 249.186 | **0.58** | **3.24E-7** | **1.95E-6** |
| FA 17:0 | [M-H]- | 269.24844 | 269.2486 | **0.79** | **1.53E-6** | **7.45E-6** |
| FA 17:1 | [M-H]- | 267.23245 | 267.233 | **0.86** | **0.029** | **0.049** |
| FA 17:2 | [M-H]- | 265.21741 | 265.21729 | **0.72** | **7.69E-5** | **2.58E-4** |
| FA 18:0 | [M-H]- | 283.2648 | 283.26431 | **0.79** | **7.13E-10** | **9.48E-9** |
| FA 18:2 | [M-H]- | 279.23328 | 279.233 | **0.72** | **4.05E-5** | **1.47E-4** |
| FA 18:3 | [M-H]- | 277.21704 | 277.21729 | **0.60** | **2.51E-6** | **1.17E-5** |
| FA 18:4 | [M-H]- | 275.20132 | 275.20169 | **0.43** | **7.18E-9** | **7.00E-8** |
| FA 19:0 | [M-H]- | 297.27979 | 297.27991 | **0.80** | **1.35E-6** | **6.72E-6** |
| FA 19:1 | [M-H]- | 295.26337 | 295.26431 | **0.80** | **0.012** | **0.022** |
| FA 20:0 | [M-H]- | 311.29541 | 311.29559 | **0.75** | **2.68E-9** | **3.02E-8** |
| FA 20:2 | [M-H]- | 307.2645 | 307.26431 | **0.81** | **0.008** | **0.015** |
| FA 20:3 | [M-H]- | 305.24835 | 305.2486 | **0.62** | **7.59E-7** | **4.16E-6** |
| FA 20:4 | [M-H]- | 303.23264 | 303.233 | **0.44** | **7.20E-12** | **2.63E-10** |
| FA 20:5 | [M-H]- | 301.21677 | 301.21729 | **0.45** | **5.47E-8** | **4.45E-7** |
| FA 22:0 | [M-H]- | 339.32736 | 339.3269 | **0.80** | **2.17E-7** | **1.44E-6** |
| FA 22:1 | [M-H]- | 337.31143 | 337.31119 | **0.49** | **0.024** | **0.041** |
| FA 22:2 | [M-H]- | 335.29605 | 335.29559 | **0.79** | **0.010** | **0.019** |
| FA 22:3 | [M-H]- | 333.27982 | 333.27991 | **0.81** | **0.011** | **0.021** |
| FA 22:5 | [M-H]- | 329.24826 | 329.2486 | **0.65** | **1.97E-5** | **7.67E-5** |
| FA 22:6 | [M-H]- | 327.23303 | 327.233 | **0.55** | **5.47E-8** | **4.45E-7** |
| FA 24:0 | [M-H]- | 367.3584 | 367.35818 | **1.01** | **0.008** | **0.017** |
| FA 24:1 | [M-H]- | 365.34256 | 365.3425 | **0.80** | **0.020** | **0.035** |
| FA 24:2 | [M-H]- | 363.32632 | 363.3269 | **0.81** | **0.003** | **0.007** |
| FA 26:0 | [M-H]- | 395.38879 | 395.3895 | **1.50** | **0.042** | **0.068** |
| FA 26:1_isomer1 | [M-H]- | 393.37378 | 393.37381 | **0.53** | **4.09E-6** | **1.80E-5** |
| FA 26:1_isomer2 | [M-H]- | 393.3739 | 393.37381 | **0.65** | **1.16E-4** | **3.70E-4** |
| FA 26:2_isomer1 | [M-H]- | 391.35757 | 391.35818 | **0.48** | **1.43E-6** | **7.08E-6** |
| FA 26:2_isomer2 | [M-H]- | 391.35928 | 391.35818 | **0.49** | **2.36E-6** | **1.11E-5** |
| FA 27:0 | [M-H]- | 409.40424 | 409.40509 | **1.65** | **0.003** | **0.007** |
| FA 28:0 | [M-H]- | 423.42096 | 423.42081 | **1.50** | **0.043** | **0.070** |
| **Lipids** | **Adduct type** | **m/z**  **(Average)** | **m/z**  **(Expected)** | **AD/N** | ***P*** | **Adj. *P*** |
| FA 28:2 | [M-H]- | 419.38943 | 419.3895 | **0.56** | **0.004** | **0.009** |
| FA 28:4 | [M-H]- | 415.35364 | 415.35818 | **0.77** | **4.20E-4** | **0.001** |
| Fatty acid transport | | | | | | |
| AC(10:0) | [M+H]+ | 316.2468 | 316.2482 | **0.33** | **1.18E-7** | **8.33E-7** |
| AC(12:0) | [M+H]+ | 344.2778 | 344.27951 | **0.36** | **1.84E-6** | **8.89E-6** |
| AC(13:0) | [M+H]+ | 358.29449 | 358.2952 | **0.66** | **0.049** | **0.077** |
| AC(14:0) | [M+H]+ | 372.31018 | 372.31079 | **0.70** | **5.03E-5** | **1.78E-4** |
| AC(14:1) | [M+H]+ | 370.29428 | 370.2952 | **0.72** | **4.62E-4** | **0.001** |
| AC(16:0) | [M+H]+ | 400.34006 | 400.3421 | **0.78** | **7.68E-4** | **0.002** |
| AC(16:1) | [M+H]+ | 398.32449 | 398.32651 | **0.87** | **0.024** | **0.041** |
| AC(18:2) | [M+H]+ | 424.34052 | 424.3421 | **0.53** | **2.95E-10** | **4.80E-9** |
| AC(20:4) | [M+H]+ | 448.33713 | 448.3421 | **0.82** | **0.046** | **0.074** |
| AC(20:5) | [M+H]+ | 446.32269 | 446.32651 | **0.53** | **1.67E-10** | **3.33E-9** |
| AC(24:0) | [M+H]+ | 512.46442 | 512.46729 | **0.54** | **1.11E-6** | **5.75E-6** |
| AC(26:0) | [M+H]+ | 540.49872 | 540.4986 | **0.78** | **0.020** | **0.035** |
| AC(26:1) | [M+H]+ | 538.48096 | 538.48297 | **0.75** | **0.015** | **0.028** |
| Cholesterol metabolism | | | | | | |
| CE(18:2) | [M+NH4]+ | 666.61438 | 666.61841 | **0.75** | **5.83E-4** | **0.002** |
| Sphingolipid metabolism | | | | | | |
| Cer(12:0;2O/22:2) | [M+H]+ | 658.64508 | 658.64972 | **0.75** | **0.001** | **0.004** |
| Cer(18:1;2O/18:0) | [M+H-H2O]+ | 548.53864 | 548.5401 | **2.18** | **7.59E-7** | **4.16E-6** |
| Cer(18:1;2O/22:0) | [M+H-H2O]+ | 604.60089 | 604.60272 | **0.86** | **0.015** | **0.028** |
| Cer(18:2;2O/22:0) | [M+H-H2O]+ | 602.58289 | 602.5871 | **0.45** | **1.72E-11** | **5.60E-10** |
| Cer(18:1;2O/23:0) | [M+H-H2O]+ | 618.61615 | 618.61841 | **0.82** | **0.010** | **0.020** |
| Cer(18:1;2O/24:0) | [M+H-H2O]+ | 632.63202 | 632.63397 | **0.84** | **0.017** | **0.030** |
| Cer(18:1;2O/24:1) | [M+H-H2O]+ | 630.6153 | 630.61841 | **0.60** | **5.47E-8** | **4.45E-7** |
| HexCer(18:1;2O/16:0) | [M+H-H2O]+ | 682.56097 | 682.56158 | **0.72** | **0.032** | **0.053** |
| HexCer(18:1;2O/22:0) | [M+H-H2O]+ | 766.6535 | 766.65552 | **0.64** | **1.33E-5** | **5.20E-5** |
| HexCer(16:0;2O/24:2) | [M+H-H2O]+ | 764.63727 | 764.63989 | **0.41** | **4.90E-6** | **2.11E-5** |
| HexCer(18:1;2O/23:0) | [M+H-H2O]+ | 780.6673 | 780.6712 | **0.62** | **5.90E-5** | **2.04E-4** |
| HexCer(18:1;2O/24:0) | [M+H-H2O]+ | 794.68164 | 794.68683 | **0.69** | **2.92E-5** | **1.09E-4** |
| SM(28:1;2O) | [M+H]+ | 619.4798 | 619.48102 | **3.74** | **0.047** | **0.075** |
| SM(28:2;2O) | [M+H]+ | 617.45667 | 617.46527 | **0.69** | **4.20E-4** | **0.001** |
| SM(23:1;2O/8:0) | [M+H]+ | 661.52429 | 661.52789 | **0.60** | **9.20E-7** | **4.87E-6** |
| SM(32:1;3O) | [M+H]+ | 691.53583 | 691.53851 | **0.82** | **0.002** | **0.004** |
| SM(18:1;2O/14:0) | [M+H]+ | 675.54108 | 675.54358 | **0.81** | **1.43E-4** | **4.44E-4** |
| SM(32:2;2O) | [M+Na]+ | 695.50793 | 695.50983 | **0.70** | **3.41E-6** | **1.54E-5** |
| SM(17:1;2O/16:0) | [M+H]+ | 689.55524 | 689.5592 | **0.79** | **9.47E-5** | **3.08E-4** |
| SM(18:2;2O/15:0) | [M+H]+ | 687.5415 | 687.54358 | **0.72** | **8.10E-5** | **2.69E-4** |
| SM(16:0;2O/18:0) | [M+H]+ | 705.58612 | 705.59052 | **0.83** | **2.86E-4** | **8.54E-4** |
| SM(18:1;2O/16:0) | [M+H]+ | 703.5719 | 703.57489 | **0.85** | **2.21E-5** | **8.43E-5** |
| SM(18:2;2O/16:0) | [M+H]+ | 701.55811 | 701.5592 | **0.85** | **1.66E-4** | **5.13E-4** |
| **Lipids** | **Adduct type** | **m/z**  **(Average)** | **m/z**  **(Expected)** | **AD/N** | ***P*** | **Adj. *P*** |
| SM(18:1;2O/16:2) | [M+H]+ | 699.54004 | 699.54358 | **0.57** | **5.87E-8** | **4.69E-7** |
| SM(18:1;2O/17:0) | [M+H]+ | 717.58826 | 717.59052 | **0.80** | **4.84E-4** | **0.001** |
| SM(18:2;2O/17:0) | [M+H]+ | 715.57263 | 715.57489 | **0.75** | **5.59E-5** | **1.96E-4** |
| SM(36:0;2O) | [M+H]+ | 733.61719 | 733.62183 | **1.84** | **1.29E-4** | **4.04E-4** |
| SM(36:4;3O) | [M+H]+ | 741.55145 | 741.55408 | **0.69** | **2.48E-7** | **1.58E-6** |
| SM(19:1;2O/17:0) | [M+H]+ | 731.60461 | 731.6062 | **1.30** | **0.032** | **0.053** |
| SM(18:2;2O/18:1) | [M+H]+ | 727.57068 | 727.57489 | **0.77** | **4.28E-5** | **1.54E-4** |
| SM(18:2;2O/18:2) | [M+H]+ | 725.55524 | 725.5592 | **0.61** | **6.60E-6** | **2.76E-5** |
| SM(18:2;2O/19:0) | [M+H]+ | 743.60559 | 743.6062 | **0.82** | **0.022** | **0.039** |
| SM(18:1;2O/20:0) | [M+H]+ | 759.6344 | 759.63751 | **0.81** | **7.87E-6** | **3.20E-5** |
| SM(18:2;2O/20:0) | [M+H]+ | 757.61768 | 757.62183 | **0.78** | **3.44E-5** | **1.28E-4** |
| SM(18:1;2O/20:2) | [M+H]+ | 755.60254 | 755.6062 | **1.33** | **0.005** | **0.011** |
| SM(38:4;2O) | [M+Na]+ | 775.56842 | 775.57239 | **1.51** | **0.001** | **0.004** |
| SM(18:1;2O/21:0) | [M+H]+ | 773.65002 | 773.65308 | **0.71** | **4.51E-7** | **2.61E-6** |
| SM(18:2;2O/21:0) | [M+H]+ | 771.6369 | 771.63751 | **0.74** | **0.001** | **0.003** |
| SM(18:1;2O/22:0) | [M+H]+ | 787.6676 | 787.66882 | **0.61** | **4.42E-10** | **6.46E-9** |
| SM(40:1;3O) | [M+H]+ | 803.66321 | 803.6637 | **0.69** | **0.002** | **0.005** |
| SM(18:2;2O/22:0) | [M+H]+ | 785.64856 | 785.65308 | **0.78** | **5.14E-7** | **2.93E-6** |
| SM(41:0;3O) | [M+H]+ | 819.69312 | 819.69501 | **0.30** | **7.39E-6** | **3.05E-5** |
| SM(17:1;2O/24:0) | [M+H]+ | 801.68372 | 801.68439 | **0.77** | **8.85E-6** | **3.53E-5** |
| SM(41:1;3O) | [M+H]+ | 817.67609 | 817.67932 | **0.59** | **1.84E-4** | **5.60E-4** |
| SM(18:2;2O/23:0) | [M+H]+ | 799.66803 | 799.66882 | **0.68** | **1.05E-6** | **5.46E-6** |
| SM(18:1;2O/24:0) | [M+H]+ | 815.69482 | 815.70007 | **0.82** | **6.22E-5** | **2.13E-4** |
| SM(42:1;3O) | [M+H]+ | 831.69122 | 831.69501 | **0.79** | **0.018** | **0.033** |
| SM(18:1;2O/24:1) | [M+H]+ | 813.68176 | 813.68439 | **0.77** | **8.85E-6** | **3.53E-5** |
| SM(18:2;2O/24:0) | [M+H]+ | 813.68207 | 813.68439 | **0.75** | **2.36E-6** | **1.11E-5** |
| SM(42:5;2O) | [M+Na]+ | 829.6178 | 829.61938 | **1.66** | **0.003** | **0.007** |
| SM(18:2;2O/25:0） | [M+H]+ | 827.69464 | 827.70007 | **0.76** | **0.005** | **0.010** |
| SM(19:1;2O/24:0) | [M+H]+ | 829.71429 | 829.7157 | **0.78** | **0.005** | **0.010** |
| Glycerophospholipid metabolism | | | | | | |
| LPC(14:0/0:0) | [M+H]+ | 468.30765 | 468.3085 | **0.28** | **6.70E-11** | **1.47E-9** |
| LPC(15:0/0:0) | [M+H]+ | 482.32291 | 482.3241 | **0.26** | **2.48E-12** | **1.36E-10** |
| LPC(16:0/0:0) | [M+H]+ | 496.33829 | 496.33981 | **0.32** | **2.08E-12** | **1.36E-10** |
| LPC(16:1/0:0) | [M+H]+ | 494.32233 | 494.3241 | **0.32** | **2.04E-11** | **5.60E-10** |
| LPC(17:0/0:0) | [M+H]+ | 510.35477 | 510.35541 | **0.23** | **2.08E-12** | **1.36E-10** |
| LPC(18:0/0:0) | [M+H]+ | 524.36987 | 524.37109 | **0.24** | **2.08E-12** | **1.36E-10** |
| LPC(18:1/0:0) | [M+H]+ | 522.35382 | 522.35541 | **0.28** | **2.08E-12** | **1.36E-10** |
| LPC(18:2/0:0) | [M+H]+ | 520.33801 | 520.33978 | **0.33** | **2.04E-11** | **5.60E-10** |
| LPC(18:3/0:0) | [M+H]+ | 518.32288 | 518.3241 | **0.18** | **6.59E-12** | **2.63E-10** |
| LPC(20:0/0:0) | [M+H]+ | 552.40149 | 552.4024 | **0.19** | **2.08E-12** | **1.36E-10** |
| LPC(20:1/0:0) | [M+H]+ | 550.38354 | 550.38672 | **0.18** | **2.27E-12** | **1.36E-10** |
| LPC(20:2/0:0) | [M+H]+ | 548.36816 | 548.37109 | **0.21** | **2.48E-12** | **1.36E-10** |
| **Lipids** | **Adduct type** | **m/z**  **(Average)** | **m/z**  **(Expected)** | **AD/N** | ***P*** | **Adj. *P*** |
| LPC(20:3/0:0) | [M+H]+ | 546.35455 | 546.35541 | **0.37** | **2.42E-11** | **6.26E-10** |
| LPC(20:4) | [M+H]+ | 544.33716 | 544.33978 | **0.44** | **1.45E-9** | **1.72E-8** |
| LPC(20:5) | [M+Na]+ | 564.30377 | 564.30609 | **0.39** | **2.17E-7** | **1.44E-6** |
| LPC(22:0/0:0) | [M+H]+ | 580.43329 | 580.43372 | **0.23** | **2.97E-12** | **1.45E-10** |
| LPC(22:1) | [M+H]+ | 578.41534 | 578.41803 | **0.16** | **2.51E-10** | **4.78E-9** |
| LPC(22:4/0:0) | [M+H]+ | 572.36963 | 572.37109 | **0.39** | **2.03E-7** | **1.39E-6** |
| LPC(22:5/0:0) | [M+H]+ | 570.3551 | 570.35541 | **0.39** | **5.61E-10** | **7.95E-9** |
| LPC(22:6) | [M+Na]+ | 590.31848 | 590.32172 | **0.45** | **9.04E-10** | **1.10E-8** |
| LPC(24:0/0:0) | [M+H]+ | 608.46228 | 608.46503 | **0.45** | **4.04E-11** | **9.34E-10** |
| LPC(24:1/0:0) | [M+H]+ | 606.44556 | 606.44928 | **0.28** | **1.87E-11** | **5.60E-10** |
| LPC(24:3/0:0) | [M+H]+ | 602.42761 | 602.41803 | **0.67** | **0.001** | **0.003** |
| LPC(26:0/0:0) | [M+H]+ | 636.49493 | 636.49628 | **0.52** | **3.41E-6** | **1.54E-5** |
| LPE(16:0) | [M+H]+ | 454.29089 | 454.29279 | **0.45** | **3.20E-10** | **4.84E-9** |
| LPE(18:0) | [M+H]+ | 482.32278 | 482.3241 | **0.34** | **7.20E-12** | **2.63E-10** |
| LPE(18:1) | [M+H]+ | 480.30679 | 480.3085 | **0.38** | **9.04E-10** | **1.10E-8** |
| LPE(18:2) | [M+H]+ | 478.29105 | 478.29279 | **0.47** | **7.25E-8** | **5.58E-7** |
| LPE(22:5) | [M+H]+ | 528.30487 | 528.30847 | **0.69** | **0.008** | **0.016** |
| LPE(22:6) | [M+H]+ | 526.29193 | 526.29279 | **0.66** | **3.63E-5** | **1.33E-4** |
| PC(16:0/16:1) | [M+H]+ | 732.55273 | 732.55377 | **1.31** | **0.006** | **0.013** |
| PC(32:2) | [M+H]+ | 730.53516 | 730.53809 | **0.69** | **0.001** | **0.004** |
| PC(32:3) | [M+H]+ | 728.521 | 728.52252 | **0.48** | **2.47E-4** | **7.43E-4** |
| PC(15:0/18:2) | [M+H]+ | 744.54883 | 744.55377 | **0.78** | **0.011** | **0.021** |
| PC(33:3) | [M+Na]+ | 764.51849 | 764.52008 | **0.55** | **7.01E-4** | **0.002** |
| PC(16:0/18:0) | [M+H]+ | 762.59674 | 762.60071 | **0.76** | **2.84E-6** | **1.31E-5** |
| PC(16:0/18:2) | [M+H]+ | 758.56805 | 758.5694 | **0.92** | **0.035** | **0.057** |
| PC(16:1/18:2) | [M+H]+ | 756.54974 | 756.55377 | **0.78** | **4.84E-4** | **0.001** |
| PC(34:4)_isomer1 | [M+Na]+ | 776.51538 | 776.52008 | **0.54** | **2.32E-7** | **1.50E-6** |
| PC(34:4)_isomer2 | [M+H]+ | 754.53674 | 754.53809 | **0.68** | **5.83E-4** | **0.002** |
| PC(34:5) | [M+H]+ | 752.51935 | 752.52252 | **0.43** | **1.35E-6** | **6.72E-6** |
| PC(35:1) | [M+H]+ | 774.59961 | 774.60071 | **0.86** | **0.017** | **0.030** |
| PC(35:2) | [M+H]+ | 772.58392 | 772.58508 | **0.71** | **3.63E-5** | **1.33E-4** |
| PC(17:1/18:2) | [M+H]+ | 770.56659 | 770.5694 | **0.61** | **2.65E-7** | **1.64E-6** |
| PC(35:4)_isomer2 | [M+H]+ | 768.55139 | 768.55377 | **0.79** | **0.016** | **0.029** |
| PC(35:6) | [M+H]+ | 764.52002 | 764.52252 | **0.95** | **0.002** | **0.005** |
| PC(18:0/18:0) | [M+H]+ | 790.63348 | 790.63202 | **0.68** | **2.65E-7** | **1.64E-6** |
| PC(18:0/18:1) | [M+H]+ | 788.61267 | 788.61639 | **0.89** | **0.049** | **0.077** |
| PC(18:1/18:1) | [M+H]+ | 786.59961 | 786.60071 | **0.77** | **7.77E-8** | **5.88E-7** |
| PC(16:0/20:3) | [M+H]+ | 784.58386 | 784.58508 | **0.69** | **1.18E-7** | **8.33E-7** |
| PC(18:2/18:2) | [M+H]+ | 782.56519 | 782.5694 | **0.39** | **1.30E-10** | **2.72E-9** |
| PC(36:5)_isomer1 | [M+Na]+ | 802.53424 | 802.53571 | **0.34** | **2.95E-10** | **4.80E-9** |
| PC(36:6) | [M+Na]+ | 800.51733 | 800.52008 | **0.64** | **3.31E-4** | **9.81E-4** |
| PC(37:2) | [M+H]+ | 800.61371 | 800.61639 | **0.60** | **2.72E-10** | **4.78E-9** |
| **Lipids** | **Adduct type** | **m/z**  **(Average)** | **m/z**  **(Expected)** | **AD/N** | ***P*** | **Adj. *P*** |
| PC(17:0/20:3) | [M+H]+ | 798.59625 | 798.60071 | **0.69** | **6.67E-7** | **3.75E-6** |
| PC(17:0/20:4) | [M+H]+ | 796.58313 | 796.58508 | **0.81** | **0.020** | **0.036** |
| PC(37:5) | [M+Na]+ | 816.54602 | 816.55139 | **0.68** | **0.001** | **0.003** |
| PC(15:0/22:6) | [M+H]+ | 792.55115 | 792.55377 | **0.69** | **0.002** | **0.006** |
| PC(38:1) | [M+H]+ | 816.64459 | 816.64771 | **0.55** | **4.24E-9** | **4.43E-8** |
| PC(38:2)_isomer1 | [M+H]+ | 814.62933 | 814.63202 | **0.46** | **3.13E-11** | **7.64E-10** |
| PC(18:0/20:2) | [M+H]+ | 814.62988 | 814.63202 | **0.79** | **0.001** | **0.004** |
| PC(38:3) | [M+H]+ | 812.61511 | 812.61639 | **0.79** | **0.002** | **0.004** |
| PC(18:1/20:3) | [M+H]+ | 810.59723 | 810.60071 | **0.79** | **0.001** | **0.003** |
| PC(18:1/20:4) | [M+H]+ | 808.58051 | 808.58508 | **0.70** | **7.29E-5** | **2.46E-4** |
| PC(38:5)_isomer1 | [M+Na]+ | 830.5647 | 830.56702 | **0.89** | **0.035** | **0.057** |
| PC(18:2_20:4) | [M+H]+ | 806.56714 | 806.5694 | **0.49** | **7.13E-10** | **9.48E-9** |
| PC(38:7)_isomer1 | [M+Na]+ | 826.53259 | 826.53571 | **0.38** | **6.18E-9** | **6.17E-8** |
| PC(38:7)_isomer2 | [M+Na]+ | 826.53259 | 826.53571 | **0.69** | **1.16E-4** | **3.70E-4** |
| PC(39:4) | [M+Na]+ | 846.59314 | 846.59833 | **0.79** | **0.001** | **0.003** |
| PC(39:5) | [M+Na]+ | 844.58002 | 844.5827 | **0.78** | **0.004** | **0.009** |
| PC(39:6) | [M+H]+ | 820.57849 | 820.58508 | **0.65** | **0.002** | **0.004** |
| PC(40:1) | [M+Na]+ | 866.65985 | 866.66089 | **0.57** | **0.003** | **0.006** |
| PC(40:2) | [M+Na]+ | 864.64172 | 864.64532 | **0.43** | **3.64E-9** | **3.90E-8** |
| PC(40:3) | [M+H]+ | 840.64136 | 840.64771 | **0.59** | **6.18E-9** | **6.17E-8** |
| PC(40:5)_isomer1 | [M+Na]+ | 858.59625 | 858.59833 | **0.80** | **0.024** | **0.041** |
| PC(18:1/22:5) | [M+H]+ | 834.59418 | 834.60071 | **0.63** | **8.63E-7** | **4.62E-6** |
| PC(40:7) | [M+Na]+ | 854.56464 | 854.56702 | **0.66** | **4.51E-7** | **2.61E-6** |
| PC(40:8)_isomer1 | [M+Na]+ | 852.5484 | 852.55139 | **0.43** | **2.72E-10** | **4.78E-9** |
| PC(40:8)_isomer2 | [M+Na]+ | 852.54736 | 852.55139 | **0.65** | **1.05E-4** | **3.39E-4** |
| PC(42:1) | [M+Na]+ | 894.69067 | 894.6922 | **0.61** | **0.005** | **0.011** |
| PC(42:2) | [M+H]+ | 870.69165 | 870.69458 | **0.44** | **1.69E-9** | **1.95E-8** |
| PC(42:3)_isomer1 | [M+H]+ | 868.67737 | 868.67902 | **0.59** | **6.76E-8** | **5.30E-7** |
| PC(42:3)_isomer2 | [M+H]+ | 868.68414 | 868.67902 | **0.36** | **3.64E-9** | **3.90E-8** |
| PC(42:4) | [M+H]+ | 866.66003 | 866.66333 | **0.80** | **3.64E-4** | **0.001** |
| PC(42:5)_isomer2 | [M+Na]+ | 886.6286 | 886.62958 | **0.57** | **0.003** | **0.007** |
| PC(42:6) | [M+H]+ | 862.63092 | 862.63202 | **0.58** | **8.99E-5** | **2.97E-4** |
| PC(20:1/22:6) | [M+H]+ | 860.61047 | 860.61639 | **0.64** | **4.09E-6** | **1.80E-5** |
| PC(44:4) | [M+H]+ | 894.6936 | 894.69458 | **0.55** | **4.13E-8** | **3.70E-7** |
| PC(44:5) | [M+Na]+ | 914.65936 | 914.66089 | **0.78** | **0.011** | **0.021** |
| PE(16:0/18:1) | [M+H]+ | 718.53693 | 718.53809 | **1.84** | **2.47E-5** | **9.34E-5** |
| PE(16:0/18:2) | [M+H]+ | 716.52197 | 716.52252 | **1.39** | **0.008** | **0.016** |
| PE(16:0/18:3) | [M+H]+ | 714.50403 | 714.50677 | **1.66** | **0.002** | **0.005** |
| PE(16:0/20:4) | [M+H]+ | 740.52173 | 740.52252 | **1.33** | **0.008** | **0.015** |
| PE(18:2/18:2) | [M+H]+ | 740.52179 | 740.52252 | **0.43** | **8.09E-7** | **4.39E-6** |
| PE(16:0/22:5) | [M+H]+ | 766.53345 | 766.53809 | **1.37** | **0.012** | **0.023** |
| PE(18:0/20:4) | [M+H]+ | 768.55164 | 768.55377 | **1.24** | **0.021** | **0.037** |
| **Lipids** | **Adduct type** | **m/z**  **(Average)** | **m/z**  **(Expected)** | **AD/N** | ***P*** | **Adj. *P*** |
| PE(38:6) | [M+Na]+ | 786.49957 | 786.50439 | **1.45** | **5.31E-4** | **0.001** |
| PE(18:0/22:6) | [M+H]+ | 792.55206 | 792.55377 | **1.53** | **0.002** | **0.004** |
| PI(34:1) | [M+NH4]+ | 854.5647 | 854.57532 | **0.63** | **1.18E-7** | **8.33E-7** |
| PI(34:2)_isomer1 | [M+NH4]+ | 852.5542 | 852.55969 | **0.77** | **0.026** | **0.045** |
| PI(36:1) | [M+NH4]+ | 882.60345 | 882.60663 | **0.45** | **9.67E-9** | **9.23E-8** |
| PI(36:2)_isomer1 | [M+NH4]+ | 880.58582 | 880.591 | **0.69** | **4.34E-6** | **1.89E-5** |
| PI(36:2)_isomer2 | [M+NH4]+ | 880.58636 | 880.591 | **0.74** | **4.84E-4** | **0.001** |
| PI(36:3) | [M+NH4]+ | 878.57184 | 878.57532 | **0.53** | **5.44E-8** | **4.45E-7** |
| PI(36:4)_isomer1 | [M+NH4]+ | 876.55914 | 876.55969 | **0.79** | **0.012** | **0.023** |
| PI(38:4) | [M+NH4]+ | 904.58502 | 904.591 | **0.81** | **0.002** | **0.004** |
| PI(38:5) | [M+NH4]+ | 902.57111 | 902.57532 | **0.49** | **3.84E-8** | **3.51E-7** |
| PI(38:6) | [M+NH4]+ | 900.55432 | 900.55969 | **0.72** | **0.008** | **0.016** |
| PI(40:3) | [M+H]+ | 939.59589 | 939.59332 | **0.90** | **0.009** | **0.018** |
| Glycerolipid metabolism | | | | | | |
| DG(32:1) | [M+H]+ | 589.47888 | 589.48022 | **0.72** | **0.038** | **0.063** |
| DG(16:0/18:1) | [M+NH4]+ | 612.55469 | 612.55621 | **0.74** | **7.68E-4** | **0.002** |
| DG(16:0/18:2) | [M+NH4]+ | 610.53931 | 610.54053 | **0.70** | **1.84E-4** | **5.60E-4** |
| DG(34:3) | [M+Na]+ | 613.47699 | 613.48022 | **0.55** | **2.32E-7** | **1.50E-6** |
| DG(35:2) | [M+Na]+ | 629.50745 | 629.51147 | **0.83** | **0.015** | **0.028** |
| DG(18:1/18:3) | [M+NH4]+ | 634.53845 | 634.54053 | **0.58** | **0.018** | **0.033** |
| DG(18:2/18:2) | [M+NH4]+ | 634.53857 | 634.54053 | **0.67** | **2.03E-4** | **6.14E-4** |
| DG(18:2/18:3) | [M+NH4]+ | 632.52411 | 632.5249 | **0.31** | **5.10E-8** | **4.45E-7** |
| DG(18:3/18:2) | [M+NH4]+ | 632.52344 | 632.5249 | **0.44** | **8.33E-8** | **6.20E-7** |
| DG(38:4)_isomer1 | [M+Na]+ | 667.52496 | 667.52722 | **0.73** | **0.001** | **0.003** |
| DG(38:4)_isomer2 | [M+Na]+ | 667.52478 | 667.52722 | **0.69** | **5.83E-4** | **0.002** |
| DG(18:2/20:3) | [M+NH4]+ | 660.55261 | 660.55621 | **0.45** | **6.60E-6** | **2.76E-5** |
| DG(38:7) | [M+Na]+ | 661.47913 | 661.48022 | **0.60** | **7.01E-4** | **0.002** |
| DG(22:1/18:2) | [M+NH4]+ | 692.61383 | 692.61877 | **1.14** | **0.012** | **0.022** |
| DG(18:1/22:5) | [M+NH4]+ | 686.57129 | 686.57178 | **0.76** | **0.026** | **0.045** |
| DG(18:2/22:5) | [M+NH4]+ | 684.55402 | 684.55621 | **0.63** | **0.009** | **0.018** |
| DG(18:2/22:6) | [M+NH4]+ | 682.53613 | 682.54053 | **0.43** | **0.007** | **0.014** |
| TG(8:0/12:0/14:0)) | [M+NH4]+ | 628.54993 | 628.55109 | **1.44** | **0.024** | **0.041** |
| TG(12:0/12:0/14:0) | [M+NH4]+ | 684.61462 | 684.61371 | **0.80** | **0.015** | **0.028** |
| TG(12:0/14:0/16:0) | [M+NH4]+ | 740.67432 | 740.67627 | **1.65** | **0.011** | **0.022** |
| TG(15:0/16:0/17:0) | [M+NH4]+ | 824.76489 | 824.7702 | **1.15** | **0.012** | **0.023** |
| TG(12:0/18:2/18:2) | [M+NH4]+ | 816.70703 | 816.70758 | **0.72** | **0.022** | **0.038** |
| TG(12:0/18:2/18:3) | [M+Na]+ | 819.64325 | 819.64728 | **0.67** | **0.005** | **0.011** |
| TG(15:0/16:0/18:1) | [M+NH4]+ | 836.76495 | 836.7702 | **1.10** | **0.027** | **0.046** |
| TG(16:0/16:0/18:0) | [M+NH4]+ | 852.79565 | 852.80151 | **1.16** | **0.047** | **0.075** |
| TG(16:0/16:0/18:1) | [M+NH4]+ | 850.7851 | 850.78583 | **1.37** | **0.001** | **0.004** |
| TG(16:0/16:1/18:1) | [M+NH4]+ | 848.7699 | 848.7702 | **1.30** | **0.004** | **0.008** |
| TG(14:0/18:2/18:3) | [M+Na]+ | 847.67426 | 847.67859 | **0.69** | **0.002** | **0.005** |
| **Lipids** | **Adduct type** | **m/z**  **(Average)** | **m/z**  **(Expected)** | **AD/N** | ***P*** | **Adj. *P*** |
| TG(14:0/18:2/18:4) | [M+Na]+ | 845.65851 | 845.66302 | **0.60** | **5.90E-5** | **2.04E-4** |
| TG(16:0/17:0/18:1) | [M+NH4]+ | 864.79944 | 864.80151 | **1.27** | **0.004** | **0.009** |
| TG(16:0/17:0/18:2) | [M+NH4]+ | 862.7829 | 862.78583 | **1.16** | **0.021** | **0.037** |
| TG(16:0/17:1/18:3) | [M+NH4]+ | 858.75098 | 858.75452 | **0.75** | **0.008** | **0.016** |
| TG(15:0/18:2/18:3) | [M+Na]+ | 861.68945 | 861.69427 | **0.60** | **2.09E-5** | **8.04E-5** |
| TG(15:2/18:2/18:2) | [M+Na]+ | 859.6767 | 859.67859 | **0.39** | **6.56E-5** | **2.23E-4** |
| TG(16:0/18:0/18:1) | [M+NH4]+ | 878.81598 | 878.81708 | **1.38** | **0.005** | **0.011** |
| TG(16:0/18:1/18:1) | [M+NH4]+ | 876.80017 | 876.80151 | **1.36** | **0.001** | **0.003** |
| TG(16:0/18:1/18:2) | [M+NH4]+ | 874.78278 | 874.78583 | **1.23** | **5.57E-4** | **0.002** |
| TG(16:0/18:2/18:3) | [M+NH4]+ | 870.75256 | 870.75452 | **0.80** | **0.006** | **0.012** |
| TG(16:1/18:2/18:3) | [M+NH4]+ | 868.73279 | 868.73889 | **0.61** | **4.09E-6** | **1.80E-5** |
| TG(16:2/18:2/18:3) | [M+Na]+ | 871.67346 | 871.67859 | **0.50** | **2.03E-7** | **1.39E-6** |
| TG(18:2/16:3/18:3) | [M+Na]+ | 869.65631 | 869.66302 | **0.37** | **1.35E-6** | **6.72E-6** |
| TG(17:0/18:0/18:1) | [M+Na]+ | 897.78461 | 897.78821 | **1.26** | **0.046** | **0.074** |
| TG(17:1/18:1/18:3) | [M+NH4]+ | 884.76532 | 884.7702 | **0.67** | **5.83E-4** | **0.002** |
| TG(17:1/18:2/18:3) | [M+Na]+ | 887.70544 | 887.7099 | **0.56** | **5.03E-5** | **1.78E-4** |
| TG(18:1/18:2/18:2) | [M+NH4]+ | 898.7879 | 898.78583 | **0.75** | **7.01E-4** | **0.002** |
| TG(54:6)_isomer1 | [M+NH4]+ | 896.76434 | 896.7702 | **0.60** | **9.47E-5** | **3.08E-4** |
| TG(18:2/18:2/18:3) | [M+NH4]+ | 894.74872 | 894.75452 | **0.47** | **5.52E-6** | **2.35E-5** |
| TG(16:1/18:3/20:4) | [M+NH4]+ | 892.73462 | 892.73889 | **0.68** | **0.006** | **0.012** |
| TG(18:2/18:3/18:3) | [M+NH4]+ | 892.73328 | 892.73889 | **0.34** | **3.24E-7** | **1.95E-6** |
| TG(18:1/19:1/18:2) | [M+Na]+ | 919.77106 | 919.77252 | **0.80** | **0.024** | **0.041** |
| TG(19:1/18:2/18:2) | [M+Na]+ | 917.7558 | 917.7569 | **0.66** | **0.003** | **0.006** |
| TG(16:0/18:1/22:4) | [M+NH4]+ | 926.81598 | 926.81708 | **1.24** | **0.042** | **0.068** |
| TG(18:2/18:3/20:4) | [M+NH4]+ | 918.7533 | 918.75452 | **0.58** | **1.23E-4** | **3.87E-4** |
| TG(18:2/18:3/20:5) | [M+Na]+ | 921.69196 | 921.69427 | **0.45** | **9.38E-6** | **3.71E-5** |
| TG(58:8)_isomer1 | [M+NH4]+ | 948.79547 | 948.80151 | **0.78** | **0.011** | **0.022** |
| TG(18:2/18:2/22:5) | [M+NH4]+ | 946.78082 | 946.78583 | **0.63** | **6.40E-4** | **0.002** |
| TG(18:2/18:2/22:6) | [M+NH4]+ | 944.76703 | 944.7702 | **0.70** | **0.025** | **0.042** |
| TG(18:2/18:3/22:6) | [M+NH4]+ | 942.75238 | 942.75452 | **0.45** | **0.002** | **0.005** |
| TG(18:1/18:2/24:4) | [M+Na]+ | 983.80426 | 983.80377 | **0.71** | **0.032** | **0.053** |
| TG(18:1/18:2/24:5) | [M+Na]+ | 981.78717 | 981.78821 | **0.63** | **0.027** | **0.045** |
| TG(18:2/20:5/22:6) | [M+Na]+ | 971.70813 | 971.7099 | **0.53** | **0.046** | **0.074** |

AD, patients with aortic dissection; N, normal controls; FA, fatty acid; AC, acylcarnitine; CE, cholesteryl ester; Cer, ceramide; HexCer, hexosylceramide; SM, sphingomyelin; LPC, lysophosphatidylcholine; LPE, lysophosphatidylethanolamine; PC, phosphatidylcholine; PE, phosphatidylethanolamine; PI, phosphatidylinositol; DG, diacylglycerol; TG, triacylglycerol. Red/blue bold fonts: significantly increased/decreased in patients with AD. The value of the raw *P* was obtained by using the 2-tailed Mann-Whitney U test, and then correlated by the Benjamini-Hochberg Correction.

**Supplementary Table 3.** Characteristic changes in total lipids in patients with AD.

| **Lipids** | **AD/N** | ***P*** | **Adj. *P*** |
| --- | --- | --- | --- |
| FA | **0.74** | **1.39E-8** | **4.51E-8** |
| AC | **0.65** | **2.50E-8** | **6.50E-8** |
| CE(18:2) | **0.75** | **5.83E-4** | **7.58E-4** |
| Cer | **0.82** | **0.002** | **0.002** |
| HexCer | **0.66** | **1.57E-5** | **2.27E-5** |
| SM | **0.78** | **2.49E-9** | **1.08E-8** |
| LPC | **0.30** | **2.08E-12** | **2.70E-11** |
| LPE | **0.44** | **9.04E-10** | **5.87E-9** |
| PC | **0.82** | **3.09E-6** | **5.75E-6** |
| PE | **1.34** | **0.002** | **0.003** |
| PI | **0.76** | **1.55E-7** | **3.35E-7** |
| DG | **0.65** | **5.20E-6** | **8.45E-6** |
| TG | 1.03 | 0.38 | 0.38 |

AD, patients with aortic dissection; N, normal controls; FA, fatty acid; AC, acylcarnitine; CE, cholesteryl ester; Cer, ceramide; HexCer, hexosylceramide; SM, sphingomyelin; LPC, lysophosphatidylcholine; LPE, lysophosphatidylethanolamine; PC, phosphatidylcholine; PE, phosphatidylethanolamine; PI, phosphatidylinositol; DG, diacylglycerol; TG, triacylglycerol. Red/blue bold fonts: significantly increased/decreased in patients with AD. The value of the raw *P* was obtained by using the 2-tailed Mann-Whitney U test, and then correlated by the Benjamini-Hochberg Correction.

**Supplementary Table 4.** Characteristic changes in the acyl chains of TGs in patients with AD.

| **Acyl chains** | **AD/N** | ***P*** | **Adj. *P*** |
| --- | --- | --- | --- |
| 8:0 | 1.27 | 0.145 | 0.165 |
| 12:0 | **0.73** | **4.84E-4** | **9.39E-4** |
| 14:0 | **0.66** | **1.73E-6** | **8.16E-6** |
| 15:0 | 0.88 | 0.167 | 0.184 |
| 15:2 | **0.39** | **1.67E-5** | **5.00E-5** |
| 16:0 | **1.15** | **1.63E-6** | **8.16E-6** |
| 16:1 | **1.10** | **0.022** | **0.035** |
| 16:2 | **0.48** | **1.21E-8** | **1.99E-7** |
| 16:3 | **0.37** | **1.63E-6** | **8.16E-6** |
| 17:0 | **1.14** | **0.006** | **0.010** |
| 17:1 | **0.64** | **1.57E-5** | **5.00E-5** |
| 18:0 | **1.29** | **0.005** | **0.009** |
| 18:1 | **1.12** | **2.36E-6** | **9.74E-6** |
| 18:2 | **0.88** | **1.29E-4** | **3.04E-4** |
| 18:3 | **0.65** | **5.86E-7** | **6.26E-6** |
| 18:4 | **0.58** | **7.59E-7** | **6.26E-6** |
| 19:1 | **0.72** | **1.66E-4** | **3.43E-4** |
| 20:4 | **0.61** | **6.22E-5** | **1.71E-4** |
| 20:5 | **0.48** | **1.40E-5** | **5.00E-5** |
| 22:4 | 1.14 | 0.056 | 0.072 |
| 22:5 | **0.63** | **9.97E-5** | **2.53E-4** |
| 22:6 | **0.64** | **0.012** | **0.021** |
| 24:4 | **0.66** | **0.049** | **0.064** |
| 24:5 | 0.60 | 0.084 | 0.103 |

AD, patients with aortic dissection; N, normal controls. Red/blue bold fonts: significantly increased/decreased in patients with AD. The value of the raw *P* was obtained by using the 2-tailed Mann-Whitney U test, and then correlated by the Benjamini-Hochberg Correction.

**Supplementary Table 5.** Characteristic changes in the acyl chains of PEs in patients with AD.

| **Acyl chains** | **AD/N** | ***P*** | **Adj. *P*** |
| --- | --- | --- | --- |
| sn-1 16:0 | **1.07** | **0.031** | **0.069** |
| sn-1 18:0 | 0.97 | 0.188 | 0.241 |
| sn-1 18:2 | **0.33** | **7.12E-10** | **6.41E-09** |
| sn-2 18:1 | **1.40** | **1.58E-4** | **7.11E-04** |
| sn-2 18:2 | 0.96 | 0.522 | 0.522 |
| sn-2 18:3 | 1.32 | 0.088 | 0.132 |
| sn-2 20:4 | **0.95** | **0.025** | **0.069** |
| sn-2 22:5 | 1.06 | 0.275 | 0.31 |
| sn-2 22:6 | **1.14** | **0.043** | **0.078** |

AD, patients with aortic dissection; N, normal controls. Red/blue bold fonts: significantly increased/decreased in patients with AD. The value of the raw *P* was obtained by using the 2-tailed Mann-Whitney U test, and then correlated by the Benjamini-Hochberg Correction.
